# Overexpression of an ethylene-forming ACC oxidase (ACO) gene precedes the *Minute Hilum* seed coat phenotype in *Glycine max*

**DOI:** 10.1186/s12864-020-07130-8

**Published:** 2020-10-16

**Authors:** Gracia Zabala, Anupreet Kour, Lila O. Vodkin

**Affiliations:** 1 Department of Crop Sciences, University of Illinois, Urbana, IL 61981 USA; 2 Present address: Robert M. Berne Cardiovascular Research Institute, University of Virginia School of Medicine, Charlottesville, Virginia 22908 USA

**Keywords:** Minute hilum, Ethylene forming enzyme, ACC oxidase, Seed development, Defective seed coat, RNA-Seq, Expression profiling, Soybean, *Glycine max*

## Abstract

**Background:**

To elucidate features of seed development**,** we investigated the transcriptome of a soybean isoline from the germplasm collection that contained an introgressed allele known as *minute hilum* (*mi*) which confers a smaller hilum region where the seed attaches to the pod and also results in seed coat cracking surrounding the hilum region.

**Results:**

RNAs were extracted from immature seed from an extended hilum region (i.e., the hilum and a small ring of tissue surrounding the hilum in which the cracks form) at three different developmental stages:10–25, 25–50 and 50–100 mg seed fresh weight in two independent replicates for each stage. The transcriptomes of these samples from both the Clark isoline containing the *mi* allele (PI 547628, UC413, *i*^*i*^ *R t mi G*), and its recurrent Clark 63 parent isoline (PI 548532, UC7, *i*^*i*^ *R T Mi g*), which was used for six generations of backcrossing, were compared for differential expression of 88,648 Glyma models of the soybean genome Wm82.a2. The RNA sequence data obtained from the 12 cDNA libraries were subjected to *padj* value < 0.05 and at least two-fold expression differences to select with confidence genes differentially expressed in the hilum-containing tissue of the seed coat between the two lines. Glyma.09G008400 annotated as encoding an ethylene forming enzyme, ACC oxidase (*ACO*), was found to be highly overexpressed in the *mi* hilum region at 165 RPKMs (reads per kilobase per million mapped reads) compared to the standard line at just 0.03 RPKMs. Evidence of changes in expression of genes downstream of the ethylene pathway included those involved in auxin and gibberellin hormone action and extensive differences in expression of cell wall protein genes. These changes are postulated to determine the restricted hilum size and cracking phenotypes.

**Conclusions:**

We present transcriptome and phenotypic evidence that substantially higher expression of an ethylene-forming *ACO* gene likely shifts hormone balance and sets in motion downstream changes resulting in a smaller hilum phenotype and the cracks observed in the minute hilum (*mi*) isoline as compared to its recurrent parent.

## Background

Soybean seed development is a complex process that initiates soon after flower fertilization. Three to 5 days after a flower is fertilized, the embryo with a globular shape is embedded in an acellular endosperm surrounded by a vascularized integument that provides the nutrition to both the embryo and endosperm while transforming itself into the seed coat. The embryo develops to reach the cotyledon phase 12 days after pollination (DAP) [1]. The endosperm begins to degenerate and the cotyledons and embryo continue to grow for another 45 days and up to a maximum fresh weight of approximately 400–500 mg for the green seed. After that time dessication and yellowing proceeds until approximately 70 DAP, depending on the variety and maturity group with mature seeds of fresh weight of approximately 200 mg and 10% moisture content.

During this process the seed coat (testa), which envelopes the cotyledons, develops from the ovule after fertilization and is essential in transferring assimilates and nutrients that allow the cotyledons to accumulate large amounts of storage proteins and oils in the soybean seed. Other roles contributed by the seed coat in legumes and other seed plants include production of phenolic compounds as flavonoids, anthocyanins, and proanthocyanidins that have functions in defense, water permeability, dormancy, and germination of the seed [2]. The hilum is the specialized area of the seed coat where the seed attaches to the pod and where nutrients are transferred from the pod to the seed. Hormones and possibly flavonoid compounds synthesized in the seed coat may also play a fundamental role in regulating communication between the seed coat and the endosperm and embryo. Mutations affecting seed coat composition, structure, or morphology would likely affect its function.

Studies of seed color mutations have provided valuable information on the spatial and temporal expression of genes encoding key enzymes of the flavonoid pathway all through the development of the soybean seed. The biosynthesis of isoflavonoids, flavonols, anthocyanins and proanthocyanidins is regulated by the expression of three independent loci *I* (Inhibitor), *R* (black seed and hilum color) and *T* (Tawny) in soybean seed coats. The *I* locus controls distribution of anthocyanin and proanthocyanidin pigments and comprises the multigenic, inverted repeat region of chalcone synthase (*CHS*) genes (*CHS1, CHS3* and *CHS4*) [3]. CHS is the first committed enzyme in the anthocyanin pathway. In its dominant alleles (*I* or *i*^*i*^), the *I* locus silences the expression of all nine *CHS* gene family via generation of short interfering RNAs (siRNAs) in tissue-, pattern-, and developmental-specific fashion in the seed coat [4–6] which results in completely yellow seed coats and hilum (*I*) or yellow seed coats with a pigmented hilum (*i*^*i*^). Rare spontaneous mutations of dominant *i*^*i*^ alleles result in recessive *i* alleles which have been shown to arise by homologous recombination events that change copy number and arrangement of the *CHS* genes and no longer produce *CHS siRNAs* from this locus, thus releasing silencing and permitting seed color on the whole seed coat [7]. The seed coat proper or hilum color of soybeans with the recessive alleles (*i*) are also influenced by the *R* and *T* loci producing black (*i R T*), imperfect-black (*i R t*), brown (*i r T*) and buff (*i r t*) seed coats. The *T* locus encodes a flavonoid 3′ hydroxylase (*F3’H*) gene, the expression of which drives the synthesis of the anthocyanin cyanidin pathway branch [8, 9]. The *R* locus encodes a R2R3-MYB transcription factor which positively regulates the expression of a UDP-glucose: flavonoid 3-O-glucosyltransferase (*UF3GT*) gene that functions in the last step of anthocyanin synthesis [10, 11].

Defective cracking of the seed coats as well as reduced pigment intensity appears to be due to an epistatic interaction between homozygous recessive *i* and recessive *t* alleles, indicating a connection between the flavonoid pathway and seed coat morphology [12]. A correlation was found between the *I* genotype and the abundance of a cell wall protein, SbPRP1 [13]. Cultivars with *I*/*I* genotypes and yellow seed have abundant soluble SbPRP1 in the immature seed coats. In contrast, self-black or self-brown isolines with the homozygous recessive *i* genotype have a decreased amount of soluble SbPRP1 in the unpigmented (green) immature seed coats. Soluble SbPRP1 protein is reduced in seed coats of both *i T* and *i t* genotypes as is its mRNA. Investigations of soybean lines with a different type of highly defective seed coat phenotype and genotype (*def*) called the “net pattern” showed that SbPRP1 might be insolubilized within the cell wall matrix of these isolines early in seed coat development [14] and they also demonstrated differential expression of proline-rich and other cell wall protein transcripts in a global expression analysis [15].

**Fig. 1:**
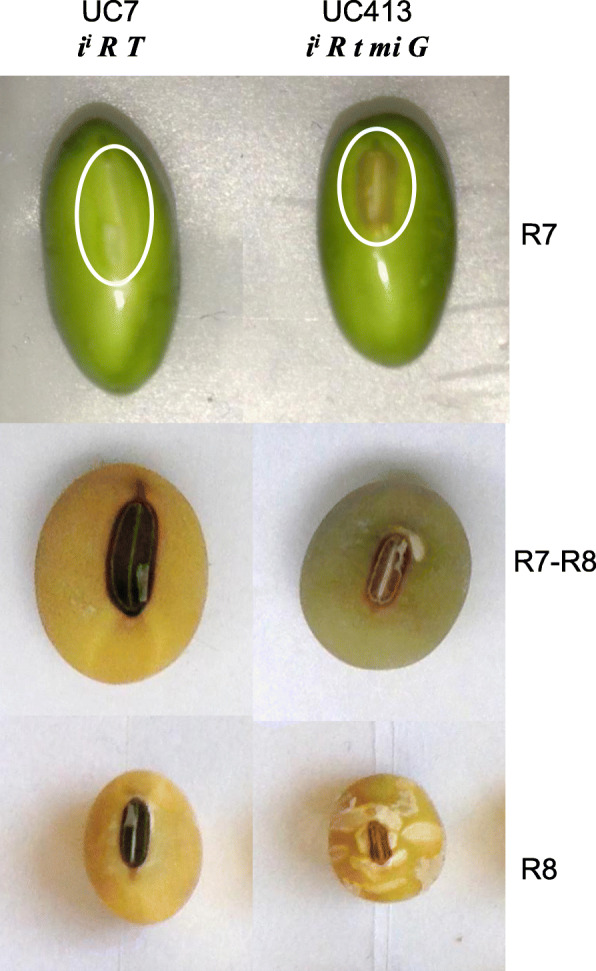
Hilum phenotypic differences between seeds of Clark isolines UC7 (*i*^*i*^ *R T Mi g*) and UC413 (*i*^*i*^ *R t mi G*) at three late developmental stages. Seeds in the left hand panels have normal size hilum (UC7) while the ones in the right (UC413) have the minute hilum phenotype and associated cracks in the seed coat. Seed coat cracks from the hilum and beyond are formed at a late stage of seed maturation and clearly seen in UC413 dry seed in the bottom right panel. The three stages of development shown to the right of the image are: full seed (R7), mid-desiccation (R7-R8) and dry seed (R8). The white oval lines around the hilum in the upper panels represent the area of the hilum and immediate seed coat region, designated the EH (extended hilum), dissected from seeds at the early maturation stages of EH10 (10–25 mg seed), EH25 (25–50 mg), and EH50 (50–100 mg) seed fresh weight developmental stages (not shown) . All genotypes are homozygous in the inbred isolines

The present study focuses on the global expression changes occurring in a morphological seed coat variant, the *minute hilum* (*mi*) allele, found in UC413 (*i*^*i*^ *R t mi G*), an isoline that results in a smaller hilum, the area of the seed coat immediately adjacent to the scar left by the funiculus that attaches the seed to its pod. In addition to the smaller size of the hilum, cracks develop in the surrounding area and the seed developmental process appears to be altered resulting in smaller seeds (Fig. 1). Since the most marked phenotype of the *mi* allele manifests itself in the hilum and surrounding area, gene expression differences in dissected portions of the seed coat containing the hilum could provide some evidence as to the underlying altered molecular components. Analysis of RNA sequence profiles from the dissected hilum and its surrounding regions from two Clark isolines, UC413 (*i*^*i*^ *R t mi G*) containing the introduced *mi* allele and the standard recurrent parent line UC7 (*i*^*i*^ *R T Mi g*) at three early stages of seed maturation (10–25, 25–50 and 50–100 mg fresh weight (fwt)), found many significantly differentially expressed transcripts. Upon careful examination of the different functional annotation classes we determined that the *mi* locus may operate through altering transcript levels of a critical enzymatic step of the ethylene biosynthetic pathway. Transcripts of an ACC oxidase (ACO), Glyma.09G008400, were the most highly differentially expressed transcripts in each of the data sets with a *padj* value of 1.9xE^− 219^ and a fold difference of 1.8 × 10^− 4^ at the earliest seed developmental stage examined. The mutant line UC413 (*i*^*i*^ *R t mi G*) with the *mi* allele has the highest level of *ACO* expression at 163 RPKMs in UC413 versus 0.03 in UC7. The ACC oxidase (previously known as ‘ethylene forming enzyme’) performs the last reaction and a key, likely rate limiting step, in the ethylene biosynthetic pathway as recently reviewed in Hoeben and Van de Paul, 2019 [16]. We pose a model whereby higher ethylene levels induce an imbalance in other hormones of the seed coat early in seed development. This model is supported by the RNA-Seq expression data revealing many differences in known downstream genes affected by ethylene action including the auxin (AUX) and gibberellin (GA) signaling pathways, as well as many changes in the cell wall category. Likely set in motion by perturbation of the ethylene pathway and other subsequent hormonal changes, these expression differences lead to the minute hilum and defective seed coat phenotype of the *mi* isoline. It is more likely that a regulator of *ACO* gene expression during hilum development, and not the *ACO* gene itself, is the *mi* locus since Glyma.09G008400 was capable of high-level expression in other non-seed coat tissues. Further, no structural changes of note were found by genomic and amplicon sequencing between the two ACO alleles of UC7 (*Mi*) and UC413 (*mi*) isoline genotypes. A small group of genes which had both expression and structural changes were determined from comparison of RNA-Seq data and structural variation of genomic data. These candidates serve as starting points to further examine this interesting, but not previously investigated, genetic trait which determines a change in the size of the hilum region during seed development and to elucidate the role of ethylene in legume seed and seed coat development.

## Results

### Genetic background and phenotypic manifestation in the seed of *minute hilum* (*mi*) gene in the UC413 Clark isoline

Figure 1 illustrates the phenotypes of the two Clark isolines, UC413 (*i*^*i*^ *R t mi G*) and it’s recurrent parent line UC7 (Clark 63, *i*^*i*^ *R T Mi g*) and Additional file 1 shows the Plant Introduction numbers, and relevant genotypes and phenotypes of the lines used in this study. All are homozygous and only one allele is shown for the genotypes. Notably, seeds of the UC413 line have a smaller hilum region. Later in development as the seeds mature and desiccate, the UC413 seed develop cracks originating at the hilum region. Both traits are associated with the minute hilum phenotype (recessive *mi* allele) which manifests in association with the recessive *t* allele. Little is known about the *mi* locus except the development of the isolines in the 1960’s by former USDA geneticist and curator of the soybean germplasm Richard L. Bernard at the University of Illinois. The history of the lines recorded in GRIN (Germplasm Resources Information Network, https://www.ars-grin.gov/) indicates that it was derived from a cross between Laredo and Harosoy. Laredo is an ancestral, un-adapted soybean line (PI 548463) with a small seed of 0.79–0.86 height-length ratio, 4.1 mg dry weight and black seed coat. It must have been the source of the *mi* allele in UC413 as Harosoy has a normal hilum size. Because of its black seed coat and tawny trichome hairs, the Laredo genotype is also *i R T*. Harosoy (PI 548573) has a larger seed of 16.1 mg dry weight with a yellow seed coat and lightly pigmented buff hilum and genotype *I r t*. It was also the source of the *t* allele in UC413 (*i*^*i*^*, R t mi G*). The line resulting from the Laredo x Harosoy cross with the minute hilum (*mi*) was back-crossed six times to the Clark 63 standard line (UC7, *i*^*i*^ *R T Mi g*), and each time the *mi* (minute hilum) and *t* (gray trichomes) phenotypes were selected to result in the UC413 (*i*^*i*^ *R t mi G)* isoline. The seeds of both lines being compared in this study, UC7 and UC413, have yellow seed coats with pigmented hila due to the *i*^*i*^ allele of the *I* locus. Those of the UC413 line have a much smaller hilum resulting from the introgressed *mi* allele in contrast with the larger black hilum of the UC7 line (Fig. 1). The UC413 seed also have a much lighter color (called imperfect black) resulting from the known epistatic interaction of the *t* allele that modifies hilum and seed coat color to reduce pigment intensity and encodes a flavonoid 3′ hydroxylase [8, 9]. The minute hilum and cracked phenotype apparently manifests only in association with the recessive *t* allele, thus indicating an epistatic interaction of the *mi* and *t* alleles. Another phenotypic difference in the seed coats of the UC413 mutant line was the stay-green seed coat trait, manifested late into the seed desiccation stage phenotype (Fig. 1), which has been attributed to the dominant allele of the *G* locus that also has yet to be identified at the molecular level.

In addition to those phenotypic differences manifested in the seed coats of the two lines, there were differences in the relative size and weight of the dried whole seed, as well as the pod size and number of seeds per pod (Table 1). The standard UC7 line seeds were larger overall and heavier compared to those of the UC413 mutant line. The dry weight of 100 seeds was consistently about two grams heavier in the standard line compared to those of the mutant line. A significant difference was also determined by the average measurement of the largest diameter of 100 seeds from each line showing that the standard seeds were two millimeters larger on average than those seeds carrying the mutant allele. A somewhat larger average pod size and number of seeds per pod of the standard line were observed from the more than 500 measurements done for each of the lines under study although these were within the standard error. The UC413 plants appeared to grow at a slower rate than those of the standard UC7 plants, resembling more the growth rate of the Laredo black progenitor.

**Table 1:** Differences between seeds from the minute hilum isoline (UC413) and the recurrent parent isoline Clark 63 (UC7)

Parameter measured	UC7 (***i***^***i***^ ***R T Mi g***)	UC413 (***i***^***i***^ ***R t mi G***)
Weight of 100 seeds^a^	23.56 ± 1.87 g	20.00 ± 1.68 g
Weight of 100 seeds^b^	16.20 ± 1.03 g	15.54 ± 0.44 g
Weight of 100 seeds^c^	14.1 g	12.1 g
Average length of 100 seeds^c^	6.57 ± 0.41 mm	4.62 ± 0.71 mm
Pod length^d^	4.55 ± 0.63 cm	3.49 ± 0.53 cm
Number of seeds per pod^d^	2.70 ± 0.58	2.15 ± 0.68

^**a**^ Weight values represent the average of 2 (UC7) and 5 (UC413) separate measurements of 100 seeds each from two different plants of each variety, made soon after harvest (6/2017)

^**b**^ Weight values represent the average of 12 (UC7) and 14 (UC413) separate measurements of 100 seeds each from two different plants of each variety, made soon after harvest (5/2016)

^**c**^ Weight and seed length of one 100 seed sample taken from seeds kept in storage for two years after harvest in 2013

^**d**^ Number of pods measured for this analysis was 547 for UC7 and 505 for UC413 from four different plants of each variety. The pod length and number of seeds per pod values are the mean of those many pod measurements

### Gene expression profiles of seed coat hilum regions from seeds of standard and *mi* isolines

Small sections of the isolated seed coat containing the hilum and immediate surrounding tissues (designated here as the extended hilum region, EH) were dissected from the rest of the seed coat as shown in Fig. 1 top panels from both standard and mutant isolines. The dissected hilum-containing seed coat sections were taken from seeds at three early to mid-maturation stages defined by different fresh weights of the whole seed and designated as EH10 (10–25 mg), EH25 (25–50 mg) and EH50 (50–100 mg). Total RNA was extracted from the collected hilum-containing seed coat sections from two independent replicates for each of the two isolines at the three seed maturation stages. A total of 12 cDNA libraries were constructed for next-generation high throughput sequencing of each transcriptome (RNA-Seq) (see Methods section). The resulting 75–100-bp RNA-Seq reads from each of the 12 libraries (Additional file 1) were aligned to the 88,647 target Glyma models of the *G. max* reference genome [17] using Bowtie v1 [18]. The numbers of RNA-Seq reads for each Glyma model were normalized in reads per kilobase of gene model per million mapped reads (RPKM) considering gene length as parameter [19].

From the total number of Glyma models in each library, the RNA-Seq RPKM values with *padj* < 0.05 were selected for statistical significance. Additionally, the ratios between the resulting RPKM values of each Glyma model from the standard (UC7) and the mutant line (UC413) of each replicate for each of the three developmental stages were calculated. The Glyma models were further filtered to obtain those with RPKM ratios > 2 and overexpressed in both of the UC7 standard line repeats or those with RPKMs ratios < 0.5 which were overexpressed in both of the UC413 isoline repeats. Finally, an RPKM selection of at > 2 RPKMs in at least one of the two UC7 repeats or in at least one of the UC413 repeats was imposed. The full list of 3782 Glyma models passing all criteria within at least one of the three stages is shown in Additional file 2. Table 2 shows that many more genes were overexpressed in the UC413 isoline containing the *mi* mutant gene than in the standard UC7 line, especially at EH10, the earliest seed development stage analyzed, where 1485 Glyma models were found to be overexpressed in the UC413 *mi* isoline but only 234 were overexpressed in the UC7 line.

**Table 2:** Number of Glyma models by gene category overexpressed in the standard UC7 and mutant UC413 (*i*^*i*^ *R t mi G*) lines at three stages of seed maturation

	UC7	UC413	UC7	UC413	UC7	UC413
**Gene categories**	EH10^*^		EH25*		EH50*	
calcium binding	0	29	2	9	1	6
carbohydrate metab.	0	49	4	14	25	26
cell division/cycle	0	44	1	7	1	22
cell wall	4	90	3	29	37	33
disease resistance	2	86	3	32	17	67
DNA replication/repair	0	3	2	1	0	8
flavonoid pathway	0	14	5	6	5	8
growth/development	1	12	8	3	2	6
heavy metal	1	13	10	5	6	5
hormone related	3	33	0	16	13	14
lipid metabolism	5	84	0	35	19	27
membrane/transporter	10	139	0	38	27	61
oxidation/reduction	32	115	23	38	42	41
photosynthesis	33	8	21	4	14	5
protein metabolism	1	129	9	28	25	54
rDNA gene	86	0	84	0	85	0
ribosomal protein	17	4	13	2	13	4
seed storage	2	13	4	4	17	6
signaling/kinases	2	59	1	6	4	23
stress/senescence	0	50	17	6	22	21
transcription factor	4	131	11	52	14	83
transcription related	6	134	6	61	13	154
other	0	15	0	5	0	12
unknown	25	231	31	74	73	173
Total per line	234	1485	258	475	475	859
Total per stage	1719		733		1334	
Total per line (−rDNA)	148	1485	174	475	390	859
Total per stage (−rDNA)	1633		649		1249	

* EH10, EH25 and EH50 represent the 10–25 mg, 25–50 mg and 50–100 mg fwt seed developmental stages, respectively

**Fig. 2:**
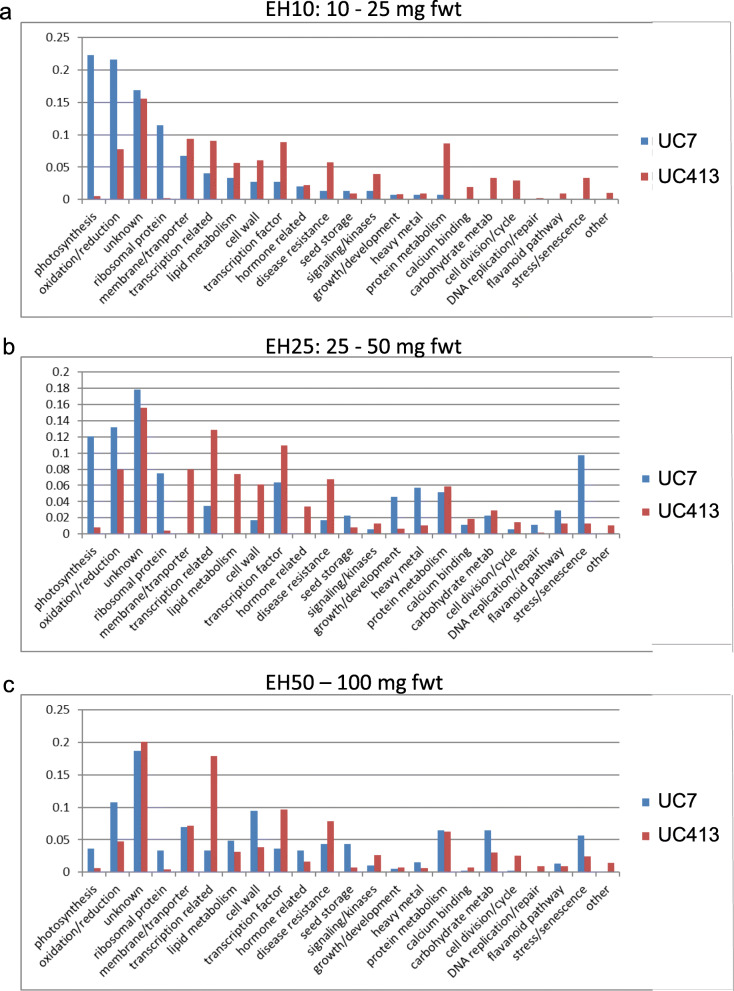
Percentages of genes by annotation categories overexpressed in the UC7 (*i*^*i*^ *R T Mi g*) (blue) or minute hilum UC413 (*i*^*i*^ *R t mi G*) (red) isolines at three seed developmental stages. **a** EH10**:** 10–25 mg; **b** EH25: 25–50 mg; **c** EH50**:**50–100 mg. The absolute number of genes in the 23 categories is shown in Table 2. The rDNA gene category has been omitted for calculation of percentages (total within a category per isoline/total overexpressed genes in the isoline)

The significantly overexpressed genes from all three stages were characterized into 24 broad groupings of annotation categories as shown in Table 2. Figure 2 displays three bar diagrams representing the percent of Glyma models within the total that were overexpressed in the standard UC7 (blue bars) and the mutant UC413 (red bars) by annotation categories for each stage. Figure 2a clearly shows that a much higher percentage of the photosynthesis, oxidation/reduction, and ribosomal protein genes were overexpressed in the standard Clark isoline UC7 in all three stages. Interestingly, the number of rDNA genes overexpressed in the standard line UC7 in all three stages (86, 84, 85) compared to none in the mutant line UC413, suggest that translation is more robust in the standard line at the three stages of seed maturation analyzed. This is supported in part by the higher number of ribosomal protein genes overexpressed in the standard line UC7. The rDNA regions are recognized due to their false annotation as Glyma models in the genome because of small interspersed protein domain fragments. The higher numbers of photosynthesis overexpressed genes in the standard line in all stages compared to those overexpressed in the *mi* line was unexpected given the fact that the seeds from the minute hilum line are as green as those of the standard line at these early seed maturation stages and stay green late into the desiccation process because of the *G* allele presence in UC413 (Fig. 1).

Most of the other categories were overexpressed in the UC413 line carrying the mutant *mi* gene in the EH10 stage. Further scrutiny of the remaining overexpressed gene categories was focused on genes whose functions may contribute more directly to the distinct seed phenotype of the mutant line UC413 and could be imparted by the unknown *mi* allele. Among those, the most relevant differences were the higher numbers of genes overexpressed in the *mi* line compared to the standard for the membrane/transporter, transcription related, transcription factor, protein/lipid/carbohydrate metabolism, and cell wall categories (Table 2, Fig. 2). Those differences in the levels of expression between the standard versus *mi* isoline are most significant at the EH10 early stage of seed development. The differences in level of gene expression between the standard and *mi* isoline decrease at the later EH50 seed developmental stage with the exception of the transcription factor and transcription related categories which remain highly over-represented in all three stages.

### The gene for an ethylene forming enzyme, *ACC oxidase* (*ACO*), is highly overexpressed in the *mi* isoline

Among the many overexpressed Glyma models in all the annotation categories, Glyma.09G008400, annotated as an ethylene forming enzyme in the hormone related category, was the most highly differentially expressed gene in the *mi* isoline UC413 with 163 RPKMs in the EH10 sample compared to only 0.03 RPKM for the UC7 recurrent parent with a fold difference of 1.8 × 10^− 4^ and highly significant *padj* value of 1.9 × 10^− 219^ (Table 3, Additional file 2). In addition, three other genes, (Glyma.15G112700, Glyma.02G268000 and Glyma.14G049500) with the same annotation were also overexpressed in the mutant line UC413 but with decreasing RPKM values of 65.8, 6.92, and 3.14, respectively, in UC413, compared to very low expression of less than 0.4 for each of the Glyma models in the UC7 standard line at the EH10 stage. Only Glyma.09G008400 and Glyma.15G112700 were also overexpressed at the EH25 and EH50 seed developmental stages (Table 3: EH25 and EH50). Additional file 3 shows the results of RNA-Seq alignments of the UC413 EH10 sample to the transcripts for each of the four *ACO* genes with no mismatches. As expected from the RPKM values Glyma.09G008400 has the most alignments. In addition, the alignments were found to coat the entire length of the transcript, indicating true expression of the gene as opposed to expression from a transposon or other repeat fragment within the gene.

**Table 3:** Ethylene related genes overexpressed in the minute hilum (*mi*) isoline UC413

Stage	Glyma model	UC7 RPKM AVE	UC413 RPKMAVE	UC7/UC413 Ratio	Gene	Category	Best Arabidopsis Annotation
EH10	Glyma.09G008400.1	0.03	**163.15**	**1.8 × 10**^**−4**^	***ACO***	Hormone	Ethylene-forming enzyme
EH10	Glyma.15G112700.1	0.03	**65.84**	**4.6 × 10**^**−4**^	***ACO***	Hormone	Ethylene-forming enzyme
EH10	Glyma.02G268000.1	0.40	**6.92**	**5.8 × 10**^**−2**^	***ACO***	Hormone	Ethylene-forming enzyme
EH10	Glyma.14G049500.1	0.29	**3.14**	**9.2 × 10**^**−2**^	***ACO***	Hormone	Ethylene-forming enzyme
EH10	Glyma.02G229400.1	1.72	**4.38**	**0.39**	***ETO1***	Signaling	Tetratricopeptide repeat
EH10	Glyma.14G197100.1^a^	1.20	**2.91**	**0.41**	***ETO1***	Signaling	Tetratricopeptide repeat
EH10	Glyma.03G191000.1^a^	1.61	**3.79**	**0.43**	***CTR1***	Signaling	Protein kinase superfamily
EH10	Glyma.19G191600.1^a^	1.70	**3.92**	**0.43**	***CTR1***	Signaling	Protein kinase superfamily
EH10	Glyma.09G002600.1^a^	2.43	**5.30**	**0.46**	***ETR1***	Signaling	Ethylene receptor 1-related
EH10	Glyma.10G188500.1	6.67	**18.71**	**0.36**	***ETR2***	Hormone	Ethylene response sensor 2 rel.
EH10	Glyma.10G008500.1	4.71	**15.01**	**0.31**	***EIN4***	Hormone	Sig trans his kin C_2_H_4_ sensor
EH10	Glyma.05G132900.1	3.66	**11.04**	**0.33**	***RAN1***	Transporter	Copper-exporting ATPase
EH10	Glyma.08G087300.1	4.02	**12.15**	**0.33**	***RAN1***	Transporter	Copper-exporting ATPase
EH10	Glyma.15G152000.1	14.00	**36.37**	**0.39**	***AP2/ERF***	T. factor	Ethylene-responsive element
EH25	Glyma.09G008400.1	0.10	**134.75**	**7.4 × 10**^**−4**^	***ACO***	Hormone	Ethylene-forming enzyme
EH25	Glyma.15G112700.1	0.07	**61.31**	**1.1 × 10**^**−3**^	***ACO***	Hormone	Ethylene-forming enzyme
EH50	Glyma.09G008400.1	0.05	**153.80**	**3.3 × 10**^**−4**^	***ACO***	Hormone	Ethylene-forming enzyme
EH50	Glyma.15G112700.1	0.07	**54.16**	**1.3 × 10**^**−3**^	***ACO***	Hormone	Ethylene-forming enzyme
EH50	Glyma.08G087300.1	9.16	**20.54**	**0.45**	***RAN1***	Transporter	Copper-exporting ATPase
EH50	Glyma.15G152000.1	27.25	**65.17**	**0.42**	***AP2/ERF***	T. factor	Ethylene-responsive element

^a^ The differential expression of these genes had *pval* < 0.05 but higher *padj* value. All others are padj < 0.05

**Fig. 3:**
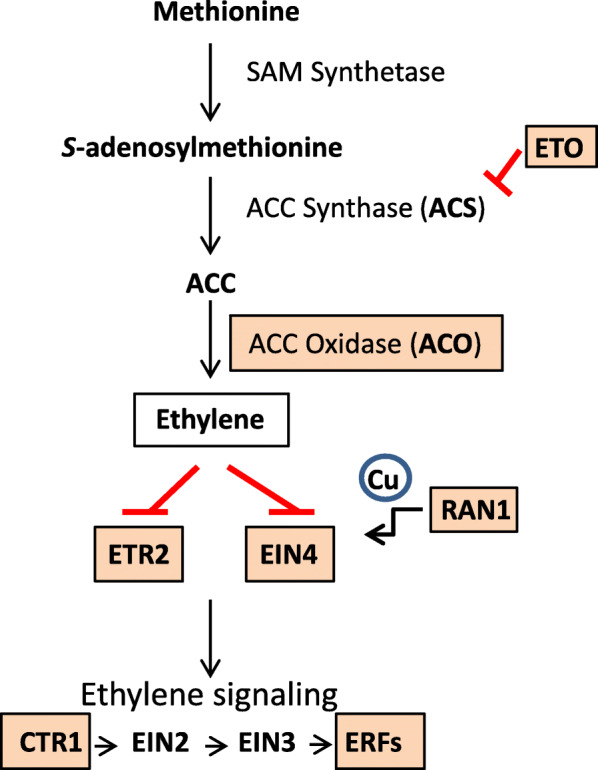
Overexpression of genes in the ethylene biosynthesis and signaling pathways in the *minute hilum* Clark isoline. The overexpressed genes in the minute hilum region of UC413 are framed and highlighted in a color background. ACC (l-aminocyclopropane-l-carboxylate); ACS (ACC Synthase); ACO (ACC Oxidase) catalyzes the last step in the synthesis of ethylene; ETO protein inhibitor of ACC Synthase; ETR2 and EIN4, ethylene receptors; RAN1 a cupper transporter; CTR1 a Raf-like kinase; EIN2 positive regulator of ethylene pathway; EIN3 transcription factor activated by EIN2; ERFs, transcription factors that turn on target genes that activate ethylene responses

The ACO enzyme is the last step in the ethylene biosynthetic pathway and currently is attributed to be the rate-limiting enzyme in the synthesis of ethylene [16]. We next examined other members of the ethylene biosynthesis and signaling pathways. As shown in Fig. 3 and Table 3, several other genes in the ethylene pathway were overexpressed in the *mi* mutant. Mutations in *ETO1*, which is a negative regulator of an *ACS5* gene (encoding ACC synthase, the first step in the pathway) induced ethylene overproduction [20, 21]. Among the overexpressed Glyma models in the seed coat samples under study, Glyma.02G229400 and Glyma.14G197100 were the only two genes with sequence similarity to *ETO1* that were differentially overexpressed in the *mi* mutant line (UC413) at the earliest stage of seed development examined, EH10 (Table 3), although Glyma.14G197100 did not meet the more stringent *padj* cutoff. Glyma models with similarity to the *Arabidopsis ACS5* and *ACS9* genes (Glyma.01G196100, Glyma.05G108900 and Glyma.11G045600) were slightly overexpressed in the EH10 hilum region of the minute hilum line UC413, but also did not pass the stringent criteria applied to the RNA-Seq data selected for the final analysis.

Once ethylene is synthesized, it enters the ethylene signal transduction pathway and among the multiple receptors/transporters identified in *Arabidopsis*, an *ETR2* (Glyma.10G188500) and *EIN4* (Glyma.10G008500) like genes [22] were found to be overexpressed in the *mi* seed coat tissue at the EH10 early stage of seed development. RAN1 is a transporter that delivers copper to the ethylene receptors and two *RAN1* like genes, Glyma.05G132900 and Glyma.08G087300, were also overexpressed in the UC413 *mi* isoline with fold differences of 0.33 (UC7/UC413 RPKMs). In addition, an ethylene-responsive AP2 transcription factor (TF), Glyma.15G152000, was also overexpressed in the mutant line with a fold difference of 0.39 (Table 3, Additional file 2).

### Auxin response genes are overexpressed in the minute hilum isoline as are several gibberellin oxidase genes

A search for AUX related genes among the hormone category of overexpressed Glyma models RNA sequences found seven *Aux/IAA* family member genes, one *DUF828* gene encoding a canalization protein containing a pleckstrin region, two *SAUR* genes coding for a SAUR-like auxin-responsive protein and a *GH3* gene encoding an auxin-responsive GH3 protein (Table 4A). Auxin promotes transcription of several classes of early genes, mainly: *AUXIN/INDOLE-3-ACETIC ACID* (*Aux/IAA*), *GRETCHEN HAGEN 3* (*GH3*), and *SMALL AUXIN UP RNA* (*SAUR*) gene family members. These are large gene families and in *Arabidopsis* there are 19 *GH3*, 81 *SAUR* and 29 *Aux/IAA* genes. AUX induces the expression of many *Aux/IAA* genes, but not all 29 members. The Aux/IAA proteins are repressors of auxin-regulated transcriptional activation and regulate auxin-mediated gene expression by controlling the activity of AUXIN RESPONSE FACTORS (ARFs) with which they heterodimerize [23, 24]. Therefore, it is possible that the 10 AUX-related overexpressed genes in the *mi* seed coat sections could interfere negatively in the AUX regulatory signaling steps during early seed development. Among the gibberellin related overexpressed genes there were two gibberellin 2-oxidase 4 (*GA2ox4*) genes, (Glyma.07G236100 and Glyma.17G037300) and a gibberellin-regulated Extensin, Glyma.02G245600 (Table 4B).

**Table 4:** Auxin and gibberellin related genes overexpressed in the *minute hilum* (*mi*) isoline UC413

Stage	Glyma Model	UC7 RPKM Ave	UC413 RPKM Ave	UC7/UC413 Ratio	Gene	Category	Best Arabidopsis Annotation
**A: Auxin related genes**							
EH10	Glyma.03G158700.1	3.85	**10.29**	**0.37**	***Aux/IAA14***	Hormone	Indole-3-acetic acid inducible 14
EH10	Glyma.05G143800.3	4.87	**12.48**	**0.39**	***Aux/IAA6***	Hormone	Auxin response factor 6
EH10	Glyma.13G127000.1^a^	4.08	**9.55**	**0.43**	***Aux/IAA11***	Hormone	Indole-3-acetic acid inducible 11
EH10	Glyma.19G161100.1	1.12	**3.29**	**0.34**	***Aux/IAA14***	Hormone	Indole-3-acetic acid inducible 14
EH10	Glyma.13G354100.1^a^	8.22	**18.17**	**0.45**	***Aux/IAA18***	Hormone	Auxin-responsive prot. IAA18
EH10	Glyma.15G012700.2^a^	34.74	**79.41**	**0.44**	***Aux/IAA15***	Hormone	AUX/IAA transcriptional regulator
EH10	Glyma.02G142600.1	10.72	**31.26**	**0.34**	***AUX/IAA***	Hormone	AUX/IAA transcriptional regulator
EH10	Glyma.01G002100.1^a^	2.72	**6.96**	**0.39**	***ARF19***	Hormone	Auxin-response factor − 19-AUX/IAA
EH10	Glyma.09G034300.1	0.06	**1.03**	**0.046**	***WRKY23***	Tr. Factor	WRKY Transcription factor 48 or 23
EH10	Glyma.03G157000.3^a^	2.62	**6.43**	**0.35**	***DUF828***	Hormone	Canalization prot. w. pleckstrin region
EH10	Glyma.07G051800.1^a^	0.85	**2.20**	**0.39**	***SAUR***	Hormone	SAUR-like auxin-responsive protein
EH10	Glyma.17G046000.1	10.43	**33.4**	**0.31**	***SAUR***	Hormone	SAUR-like auxin-responsive protein
EH10	Glyma.01G190600.1^a^	2.18	**3.98**	**0.55**	***GH3***	Hormone	Auxin-responsive GH3 protein
EH10	Glyma.05G101300.1	0.55	**1.88**	**0.29**	***GH3***	Hormone	Auxin-responsive GH3 protein
EH10	Glyma.19G128800.1^a^	**2.26**	1.64	**1.38**	***PIN1***	Transport	Auxin efflux carrier component 1
**B: Gibberellin related genes**							
EH10	Glyma.07G236100.1	0.38	**3.79**	**0.10**	***GA2ox4***	Hormone	Gibberellin 2-oxidase 4
EH10	Glyma.17G037300.1	0.24	**5.75**	**0.04**	***GA2ox4***	Hormone	Gibberellin 2-oxidase 4
EH10	Glyma.02G245600.1	0.03	**3.02**	**0.01**	***GAST1***	Hormone	Gibberellin-regulated prot; Extensin
EH25	Glyma.07G236100.1	0.47	**2.33**	**0.20**	***GA2ox4***	Hormone	Gibberellin 2-oxidase 4
EH25	Glyma.17G037300.1	0.14	**3.02**	**0.05**	***GA2ox4***	Hormone	Gibberellin 2-oxidase 4
EH25	Glyma.02G245600.1	0.03	**1.83**	**0.02**	***GAST1***	Hormone	Gibberellin-regulated prot; Extensin

^a^ The differential expression of these genes had *pval* < 0.05 but higher *padj* value. All others are padj < 0.05

### Cell wall related genes preferentially overexpressed in the minute hilum line UC413 (*i*^*i*^*R t mi G*) or the standard UC7 (*i*^*i*^*R T Mi g*)

**Fig. 4:**
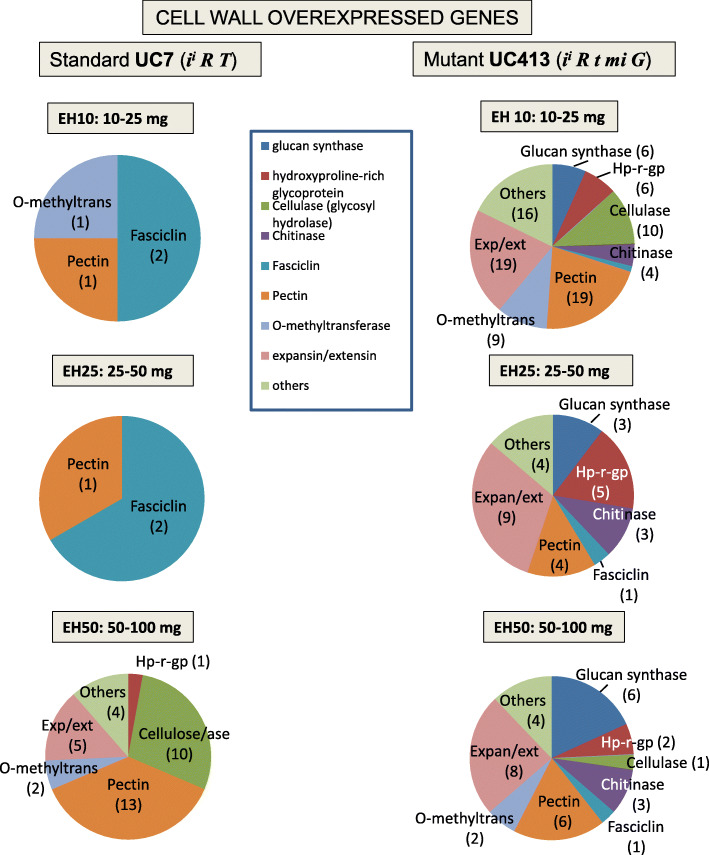
Comparison of numbers of overexpressed cell wall genes by category in the standard UC7 (*i*^*i*^ *R T Mi g*) and minute hilum UC413 (*i*^*i*^ *R t mi G*) Clark isolines. From top to bottom are the three seed maturation developmental stages measured EH10, EH25 and EH50 for the extended hilum region of the seed coats. The numbers of genes are in parentheses

In the general overview of differentially overexpressed genes at the earlier stages of seed development within the RNA-Seq of the EH10 and EH25 samples, one salient observation was the much larger number of cell wall related genes (90 and 29, respectively) overexpressed in the UC413 *mi* isoline versus only 4 and 3 in the UC7 line. Interestingly, those striking differences were lost in the latest seed stage tested, EH50, where 33 cell wall related genes were overexpressed in the hilum region of the *mi* isoline versus 37 in the standard line (Table 2). The pie charts in Fig. 4 show the distribution by categories of cell wall overexpressed genes in the two isolines with the most remarkable differences being the larger number of overexpressed genes in the glucan synthase, hydroxyproline-rich glycoprotein, cellulose/ase (glycosyl hydrolase), chitinase, pectin/ase and expansin/extensin categories in the extended hilum-containing tissue of the *mi* isoline (UC413) at both of the earliest seed developmental stages. With the exception of one pectin gene no other gene, from the five other categories mentioned above, were overexpressed in the UC7 hilum region at those two stages of seed development. Interestingly, most of those overexpressed genes in the corresponding hilum region have been described as members of cell wall loosening gene families (expansins, chitinases, glycosyl hydrolases, polygalacturonases, pectin lyases, pectin methyl esterases and pectin acetyl esterases) [25].

In contrast to the low level of overexpression of cell wall genes in the standard seed coat tissue sections at the EH10 and EH25 developmental stages, at the EH50 mg stage many more genes in the cellulose/ase (10) and pectin/ase (13) categories were overexpressed in the standard UC7 line, whereas only one gene in the cellulose/ase and six in the pectin/ase categories were overexpressed in the *mi* isoline, UC413. Also, none of the ten cellulose/ase genes overexpressed in this later stage of seed development in the standard UC7 line corresponded to those overexpressed at any of the two stages (EH10 and EH25) of seed development in the mutant line. Likewise, none of the genes overexpressed in the pectin/ase and the expansin/extensin categories in the standard line were the same as those overexpressed in the mutant line at any of the three stages of seed development examined. However, when comparing the genes overexpressed in the expansin/extensin category between the three stages of seed development in the mutant line, eight of the expansin genes were overexpressed in the two earlier stages tested (Additional file 4) and four of the expansin genes overexpressed in the EH10 stage were also overexpressed in the EH50 stage. Two of those four were overexpressed in all three stages of seed development tested.

The highest expression of the differentially expressed cell wall proteins is exhibited by Glyma.11G154900 (annotated as extensin, PRP gene*, SbPRP3*) at 1005 RPKMs in the older EH50 stage of the standard UC7 line and is reduced to 288 RPKMs in the minute hilum line at that stage. *SbPRP3* codes for one of three hydroxyproline-rich proteins characterized in soybean [13, 26]. Three other of the gibberellin-regulated extensin genes (Glyma.04G179500, Glyma.06G185300 and Glyma.14G087200) were overexpressed at the EH10 seed stage in the standard UC7 line, and Glyma.04G179500 was overexpressed at the EH25 and EH50 seed developmental stages as well (Additional file 4). However, those three genes were not overexpressed in the mutant line at any of the three stages of seed development studied. Thus, the low expression of these cell wall proteins as well as that of *SbPRP3*, Glyma.11G154900, which is expressed at high level in the standard at the EH50 stage (Additional file 4), together with the higher expression of many genes in the loosening cell wall category, could potentially influence the susceptibility of the seed coat to cracking in the minute-hilum seeds during the desiccation stage.

**Fig. 5:**
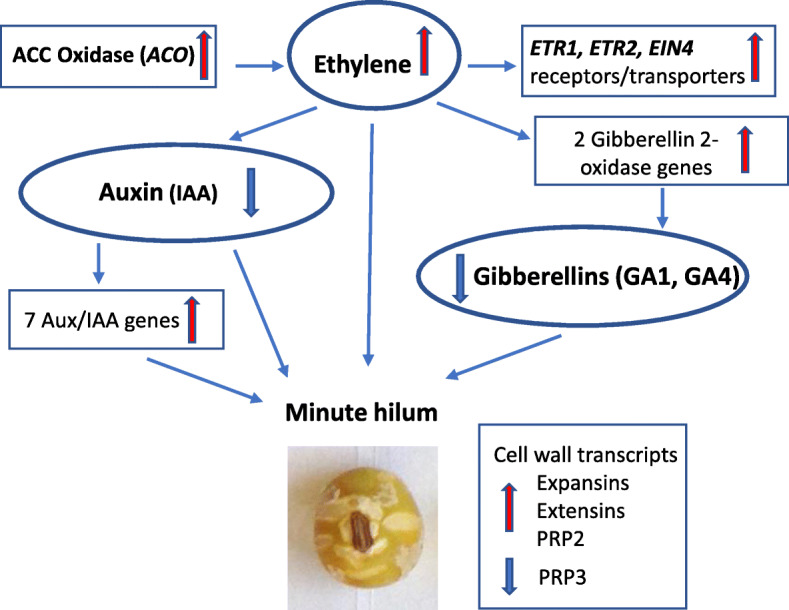
A model for altered hormone responses leading to the minute hilum trait based on global expression changes between the *minute hilum* UC413 (*i*^*i*^ *R t mi G*) and standard UC7 (*i*^*i*^ *R T Mi g*) isolines. Thick red arrows indicate upregulation and thick blue arrows indicate downregulation in the *mi* isoline UC413 of the indicated transcripts (boxed) or hormones (circled)

A proposed model showing these experimentally determined changes in gene expression in the synthesis of ethylene pathway and the downstream effects on the auxin and GA pathways, and cell wall transcripts based on known literature is diagrammed in Fig. 5 and will be elaborated further in the Discussion section.

### Expression differences between the standard and minute hilum seed coat regions of transcription factors or other genes known to promote or repress seed size

As noted in Table 2, large numbers of transcription factors were overexpressed in all three stages of the UC413 *mi* line. Additional file 5 shows a pie chart breakdown of the annotations of the transcription factor categories. The 45 MYB, 33 WRKY, and 25 AP2 subcategories were highly represented in the minute hilum tissue of UC413 whereas only 3 MYB, 1 WRKY, and 13 AP2 factors were overexpressed in the UC7 recurrent parent isoline.

Because of the smaller seed size difference in the *mi* isoline, we also examined 21 homologs (Additional file 6) of some genes reported in the literature to affect seed size or morphology in various plant systems [27–31]. These include two transcription regulator genes, *BS1* (Glyma.10G244400) and *BS2* (Glyma.20G150000) have been reported to negatively control seed, pods and leaf size in soybean [27]. Neither of those two genes was differentially expressed in the extended hilum region of the two lines under study here at any of the three seed developmental stages analyzed (Additional file 6). Neither were the soybean orthologs to *Big Grain 1* (*BG1*) from rice [28].

Because of the significant role seed coats play in the proliferation and cellularization of the endosperm and that multiple studies have shown that flavonoids synthesized in the inner most layer of the seed coat are efficient regulators of auxin transport, it was relevant to examine if any of those genes have altered expression in the soybean minute hilum seed coats. A few of those examined showed differential expression as *GmPin2b* and *PGB4* homologs that are approximately 3-fold higher in the mutant (*mi*) hilum regions. Of other putative genes encoding maternal factors, DA1 and AP2 are negative regulators restricting seed size and both are overexpressed a few fold in the hilum region of the minute hilum isoline. The putative *TTG2,* and *P450/CYP78A5/KLU*-like genes that positively regulate seed size in *Arabidopsis* [29–31] were overexpressed in the *mi* isoline that has the smaller seed. Interestingly, other P450 orthologs to Cyp78A6-like, found to increase seed size in soybean [30], were overexpressed by 3 to 4.5 fold in the standard UC7 hilum region, which has larger seeds (Additional file 6). The function of all these regulatory genes is complicated by the fact that they work in complexes to promote or restrict cell growth and differentiation as well as the fact that there are multiple family member genes encoding similar proteins that may or not be fully active to perform the specific function in the seed coats at the developmental stage under study here.

### Expression of 11 *ACO* gene family members in soybean

**Fig. 6:**
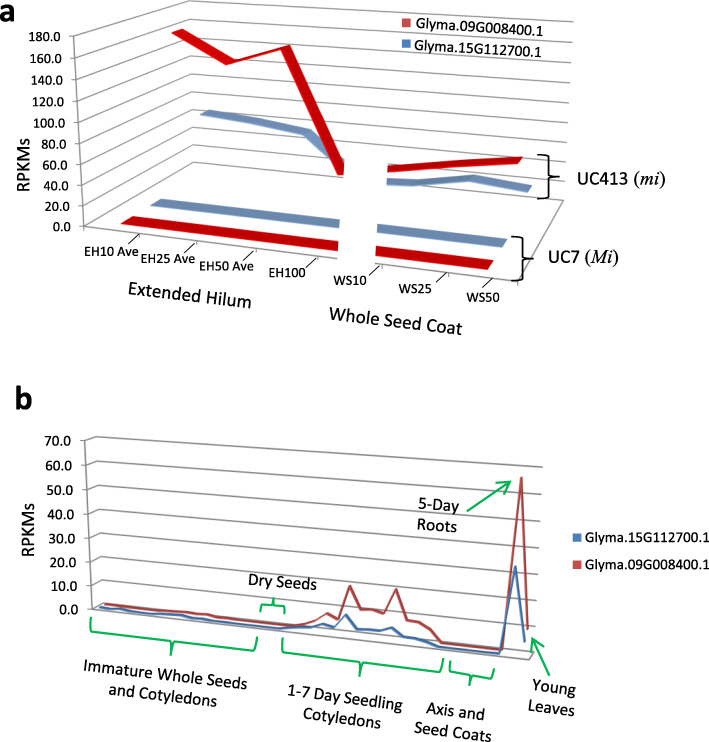
Comparative expression of *ACO* transcripts. (a) Illustration of differential expression of the two most closely related *ACO* Glyma models (red: Glyma.09G008400.1 and blue: Glyma.15G112700.1) in the RNA-Seq of the extended hilum regions (EH) or whole seed coat samples (WS) during the indicated developmental stage of either the recurrent parent isoline UC7 *(Mi)* or the minute hilum isoline UC413 *(mi)*. The average of the RPKMs for two replicates is graphed for the EH10, EH25, and EH50 data. The full data set is shown in Additional file 8a. (b) Graph of RPKMs values of each of the two *ACO* Glymas from a set of 39 total RNA-Seq libraries representing a range of developmental stages and tissues from the Williams cultivar. See Additional files 8 b,c for the full data of 11 *ACO* genes and more description of the libraries

The global view of expression changes implicates an increase in ethylene synthesis because of dramatically increased expression of the *ACO* gene encoded by Glyma.09G008400.1. A search of the annotations of all 54,064 Glyma models (excluding splice variants) of the Wm82.a2 reference soybean genome revealed 11 apparently full-length genes with annotation as ACO or ethylene forming enzyme, four of which are the ones showing differential expression between the UC7 (*Mi*) recurrent parent and minute hilum UC413 (*mi*) isolines as shown previously in (Table 3). From alignments of the coding regions using the MUSCLE program, Glyma.15G112700.1 was 95% identical to Glyma.09G008400.1 whereas Glyma.02G268000.1 and Glyma.14G049500.1 were more distant at 71 and 72%, similar respectively to Glyma.09G008400.1 and all four showed significant similarity to the known *ACO* genes in tomato by alignment of coding regions (Additional file 7). Expression data for all 11 *ACO* genes is shown in Additional file 8a and Fig. 6a shows the expression patterns of the two most highly expressed *ACO* genes in the UC413 (*mi*) minute hilum region (Glyma.09G008400.1 and Glyma.15G112700.1) compared to their very low levels of expression in the recurrent parent line UC7 (*Mi*). In addition to the EH10, EH25, and EH50 stages (for which the average of two replicates are plotted), we also sequenced RNAs from whole seed coats at the corresponding seed developmental stages, WS10, WS25, and WS50. Although there was only one replicate for each of these whole seed coat samples, they clearly showed that the whole seed coat exhibited only about 25–30% of the level of *ACO* Glyma.09G008400.1 expression as compared to the extended hilum region (for example 42 RPKMs in WS10 versus 162 in EH10). Thus, it is clear that these two Glymas were expressed more highly in the hilum region and were diluted in abundance in the whole seed coat samples. Also shown is one RNA-Seq sample from the next seed weight range EH100 (100–200 mg) which is still within the mid-maturation phase of immature green seed. In the older EH100 sample, the expression of both Glymas was only between 10 to 20% of that found in the earlier stages, indicating declining gene activity as the seed coats develop further.

Although the only RNA-Seq data available from the UC413 *mi* isoline is that reported here on extended hilum and whole seed coats, there are many RNA-Seq samples available from the reference genome cultivar, Williams 82 or from Williams, its parent isoline. We examined the expression of these 11 *ACO* Glyma models from our previously published RNA-Seq data representing a total of 39 RNA-Seq libraries constructed from a variety of development stages of whole seeds, immature cotyledons, seedling cotyledons, as well as seed coats, roots and leaves from the Williams cultivar [7, 32, 33]. As shown in Fig. 6b and Additional file 8b and 8c, the *ACO* Glyma.09G008400.1, is not expressed in any of the Williams immature whole seed or cotyledon samples, but is expressed at approximately 5–20 RPKMs in the young seedling cotyledons from about 3 to 7 days post-imbibition and in young leaves, and is highest at about 70 RPKMs in the 5-day seedling roots. These data indicate that the *ACO* Glyma.09G008400.1 was capable of being expressed in alternative tissues. Its high level of overexpression in the young immature seed coat is only found in the UC413 line, indicating either a change in the promoter region or in the regulation of the *ACO* Glyma.09G008400.1. Some of the other *ACO* Glymas, for example Glyma.02G268000.1 and Glyma.14G049500.1, rise dramatically in expression in 6 to 7-day seedling cotyledons to levels up to 400 RPKMs (Additional file 8b).

### Genomic sequence differences in the *ACO* Glyma.09G008400 between the standard UC7 *(i*^*i*^*R T Mi g)* and the minute hilum UC413 *(i*^*i*^*R t mi G)* isolines

**Fig. 7:**
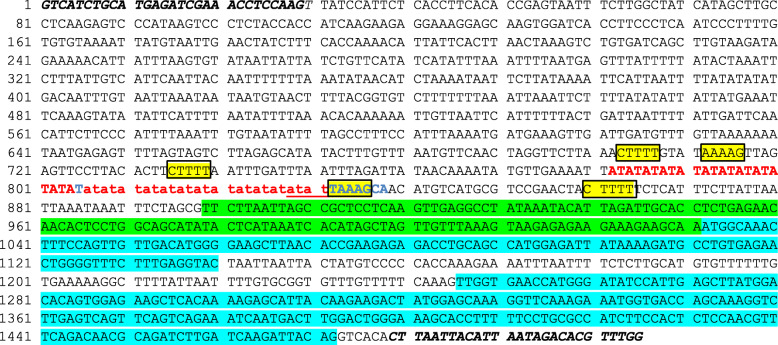
A representation of the promoter region of Glyma.09G008400.1 and the differences in amplicons from three different soybean lines. The 1505 bp sequence shown is that of the Williams 82 reference genome. Bases in bold italics at the ends are the primers used. Green highlighting indicates the 5’UTR region in the gene model, blue highlighting denotes the first two exons. The box and yellow highlight mark the five different WAAAG elements in the promoter regions of both strands. The 50 bp in red font indicate the repetitive AT region in the promoter. Within this region are the observed differences of the three sequenced amplicons as clearly shown in the alignments of Additional file 10. Both UC7 (*i*^*i*^ *R T Mi g*) and UC413 (*i*^*i*^ *R t mi G)* are identical and both lack 26 nucleotides of the AT region that are in red small letters. Amplicon sequencing of the cultivar Williams, the recurrent parent of the reference genome Williams 82 lacks 4 of the bases in the AT rich region which are also underlined in red. The bases in blue font mark the site of indel variants called by the VCF file of the whole genome sequence (Additional file 9), but they do not indicate the exact size of the deletions found in the AT-rich region as determined definitively by amplicon sequencing (Additional files 10 and 11)

Since the ACC oxidase Glyma.09G008400 was very highly overexpressed in the minute hilum UC413 isoline compared to the very low expression in UC7, the genomic sequences of that gene in the two lines genomic libraries were compared to the same gene in the reference genome using Bowtie 2 [34]. The construction of the whole genome sequencing libraries and tools used for their analysis are described in the Methods section. The SNPs (single nucleotide polymorphisms) and small indels (insertions and deletions) detected between the UC7 and UC413 alleles based on whole genome sequence are shown in Additional file 9. Several differences were predicted from the VCF (variant call format) between the standard and mutant genomic sequences for the Glyma.09G008400 gene. No changes were found in the coding sequence regions but there was an apparent small indel (insertion or deletion) found within 1 kb of the promoter region. It was also evident from the VCF file and browser displays that alignments to this region were lower depth than for the other surrounding regions. To determine the exact nature of this indel, we used high throughput amplicon sequencing to sequence a 1505 bp region surrounding this change to definitively pinpoint any changes in the promoter region. As shown in Fig. 7 and Additional file 10, the only change from the reference genome was in a string of 50 bases consisting of AT dinucleotides that are 117 bp upstream of the 5’UTR and 250 bases from the ATG start site of Glyma.09G008400 in the Williams 82 reference genome. Both the UC7 (*Mi*) and UC413 (*mi*) isolines are identical in this region and both lack 26 of these AT repeats compared to the reference Glyma.09G008400. A whole genome sequence of Williams (the recurrent parent line of the Williams 82 reference genome) differs in lacking a four base ATAT segment as compared to the reference genome. Spanning this region in the whole genome shotgun with proper alignments leads to lower base representation. However, in the targeted amplicon sequencing using high throughput sequencing, the base representation is very high at 2000 to 5000 per base as shown in Additional file 11 presenting the amplicon sequence analyses for each of the three lines. Thus, the amplicon sequence is more accurate than the alignments of whole genome data especially near regions that are hard to sequence and assemble as are strings of AT bases.

Using a motif searching engine for plants (NSITE-PL, http://linux1.softberry.com), it was determined that this AT rich region abuts the WAAAG motif **(**T/A AAAG) at position 916 of the promoter positive strand. Both isolines have five WAAAG elements (including occurrences on both strands within 203 nt upstream of the 5’UTR. The WAAAG motif [35, 36] is recognized by DOF family proteins and interestingly has been shown to work in clusters to control gene expression [37]. However, since both *Mi* and *mi* isolines have the same sequence, the changes in the surrounding AT rich element is unlikely to be the prime reason for the change in expression of the *ACO* gene between the two lines.

To confirm that the UC7 and UC413 isolines differed at the *T* locus which affects pigmentation of both the trichome hairs and the seed coat, we examined the genomic sequence around Glyma.06G202300 encoding the *F3’H* gene known to be the *T* locus [8, 9]. As shown in the genome browser displays of Additional file 12, the UC413 isoline does indeed contain the characteristic C deletion found in many gray trichome varieties (homozygous recessive *t* genotype) that introduces a premature stop codon in exon 3 of the F3’H nascent protein. We also verified the effect of the *t* allele on expression of the encoded *F3’H* gene in the RNA-Seq samples from the hilum regions of the two isolines. As shown in Additional files 2 and 6, expression of the *F3’H* gene (Glyma06G202300.1) was reduced from 108 RPKMs in the standard black hilum UC7 line to 42 RPKMs in the UC413 line which carries the *t* allele for imperfect black hilum in addition to the minute hilum *mi* allele. It is known that a premature termination codon in the recessive *t* allele, reduces but does not eliminate gene expression of the *F3’H* gene [8, 9]. Thus, the *t* allele was retained through selection for gray trichome phenotype from the original Harosoy x Larado cross. In contrast, as also shown in Additional file 12, the *R* allele was introduced into the UC413 isoline (*i*^*i*^ *R t mi G*) from the UC7 (*i*^*i*^ *R T Mi g*) recurrent parent and it carries an intact myb gene, Glyma.09G23510, as opposed to having the *r* allele from Harosoy that showed a C deletion in Exon 2 of Glyma.09G23510. This particular deletion is one of several that occur in various genotypes that are homozygous for the recessive *r* allele [10].

### Correlation of whole genome structural variation within protein coding regions with differential expression in the standard UC7 *(i*^*i*^*R T Mi g)* and the minute hilum UC413 *(i*^*i*^*R t mi G)* isolines

Finally, we extracted a list of all the genomic variants present in the UC7 and UC413 isolines relative to the reference genome. After finding and discarding the variants that were in common between the two lines, we accumulated a file of 171,389 positions having mismatched variants between the UC7 and UC413 isolines that were located either inside the Glyma models or within a 1-kb threshold on either side of the model position, *ie*., the 5′ or 3′ flanking regions. As shown in Tables 5, 70,776 variants affected 14,740 Glyma models in the UC7 line and 100,613 mismatched variants were associated with 16,146 Glyma models in the UC413 line. Next, we matched and selected those variants to include only the Glyma models that showed differential gene expression during the EH10 stage of development. As shown in Table 5, this resulted in 2966 and 4108 mismatched variants representing 560 and 607 Glyma models, respectively, in which the variant occurred within the Glyma model or a 1 kb region on either side of it in the UC7 or UC413 isolines. Further selection for the variants that would be most impactful to the protein structure were found by selecting those that occur only within the exonic coding regions and that number falls to 103 and 115 Glyma models, respectively. Selecting for small indels reduces the number to only 23 and 19 unique Glyma models, respectively. These models are potentially proteins with major disruptions as a reason for the effect on gene expression and therefore are candidates for the *mi* locus, or possibly for the *G* locus that also varies between these two isolines. Interestingly, only nine unique Glyma models had consistent expression changes in all three developmental stages (EH10, EH25, and EH50). Of the six that contained indels within the coding sequences of the UC413 alleles (Additional file 13) some were transcription factors. The one with the most significant expression changes was Glyma.14G172200.1, annotated as a Vq protein, which was consistently 7.5 to 11-fold lower in the UC413 (*i*^*i*^ *R t mi G*) isoline with a *padj* value of < 10^− 13^. Vq proteins are a class of plant specific transcription regulators involved in various growth and development processes. Of course, this approach identifies only those genes with protein variation and not promoter defects or other mechanisms that could affect expression. Nor would the approach of correlating structure and expression pinpoint candidates with changes in the active site or protein modifications that do not affect gene expression levels. Unfortunately, there are no map position data for this unusual minute-hilum trait that could help narrow the affected region. The isoline cross, which was made in the 1960’s before the era of molecular markers, was to a non-adapted variety with several other major alleles, as *G* and *t* for example, that were also introgressed at the same time. This necessarily brought in large blocks of variation in these regions and makes it difficult to pinpoint a single localized high SNP region that could be associated with the *mi* allele.

**Table 5:** Matching of whole genome and RNA-Seq data from standard and minute hilum isolines

Isoline	UC7		UC413		Category Explanation
	***i***^***i***^ ***R T Mi g***		***i***^***i***^ ***R t mi G***		
Category	Variants	Glymas	Variants	Glymas	
1	70,776	14,740	100,613	16,146	Total mismatched variants & affected Glymas
2	2966	550	4108	607	Total within or near EH10 DEGs
3	244	103	275	115	Total within exons of EH10 DEGs
4	31	23	27	19	Indels within exons of EH10 DEGs
5	6	5	7	5	Indels within exons of EH10–25-50 DEGs

Full data set of the 171,389 mismatched variants from the VCF file is shown in Additional file 12. Variants are those that differ at the same genome position number between UC7 and UC413. Thus, they do not include variants that differ from the reference genome but are identical in UC7 and UC413. Glymas are the number of unique Glymas found in the indicated set. Category explanations are found by successive filterings of the original VCF or expression data. DEGs, differentially expressed genes in stage EH10 or in all three stages EH10, EH25, and EH50 at *padj* < 0.05

## Discussion

### Numerous hormone related and cell wall gene expression changes are prominent in the minute hilum (*mi*) isoline

The recessive ***mi*** allele (Fig. 1) appears to restrict hilum growth (or cause premature maturation) and leads to a smaller hilum, smaller seed size, and defects in the seed coat that appear to emanate from the hilum region. Nothing is known about the *mi* allele in the literature except a 1960 abstract [38] and the presence of an isoline developed by the geneticist Richard L. Bernard and entered into the Soybean Germplasm collection that is maintained by the USDA (Additional file 1). Our goal was to identify gene expression variation in the extended hilum region of the seed coat brought about by the minute hilum allele (*mi*) in Clark isoline UC413 (*i*^*i*^ *R t mi G*) compared to that of the Clark 63 standard UC7 (*i*^*i*^ *R T Mi g*). Only 234, 258 and 475 genes respectively were overexpressed in the standard UC7 (*i*^*i*^ *R T Mi g*) line at the EH10, EH14, and EH50 stages, respectively, whereas a much larger number of genes (1485, 475 and 859) were overexpressed in the minute-hilum isoline UC413 (*i*^*i*^*,R,t,mi,G*) (Table 2; Additional file 2). The most striking differences were the larger numbers of genes in several categories including transcription factors, transcription related genes, hormone related, cell wall, and protein metabolism genes that were overexpressed in the *mi* line compared to those in the standard, especially at the earliest stage of development (Table 2).

On the other hand, more photosynthesis related genes were overexpressed in the standard line compared to the *mi* line at each of the three developmental stages examined. This fact suggests that even though the seed coats of the mutant line were green and indistinguishable by eye from those of the standard line at all three seed coat maturation stages analyzed, a certain number of photosynthesis related genes encoding, mostly Photosystem I psaA/psaB protein and Photosystem II reaction center proteins, are either suppressed, or out of phase, in their expression in the hilum-containing seed coat sections from the *mi* isoline relative to that of the standard line. Of the few photosynthesis related genes overexpressed in the *mi* isoline, four of them were overexpressed at the EH10 and EH25 seed developmental stages and only one, a putative chloroplastic DNA binding protein Glyma.05G076800), was overexpressed at all three stages tested (Additional file 2).

Little is known about the *G* locus, which also varies in genotype between these two lines, except that it keeps seed coats green until maturity and that a putative map position placed it near the beginning of chromosome 1 (information at Soybase, https://soybase.org/). Some of the many transcription factors which are differentially expressed could affect the stay green phenotype of the seed coat and potentially be candidates for the *G* locus function. A few of these are located near the putative map position at the beginning of Chromosome 1 (Additional file 2). The *T* locus which affects pigmentation on both the trichome hairs and the seed coat also varies between these isolines in phenotype. It is known to encode the abundant *F3’H* transcript found in the seed coats [9]. We verified both the *t* and *R* genotypes in the UC413 line by the whole genome resequencing data for these alleles (Additional file 12).

### A model for altered hormone responses leading to the minute hilum trait based on global expression changes between the *mi* and standard isolines

Although essentially no studies have been conducted on the *mi* trait except the development of an isoline carrying the gene, our RNA-Seq high throughput data brought to prominence the differential expression of an ethylene-forming enzyme ACC Oxidase (*ACO*) gene, Glyma.09G008400 that catalyzes the final step in the biosynthesis of ethylene and is also now thought to be a rate limiting step in many circumstances [16]. ACC Synthase (ACS), which acts immediately upstream in the pathway, had previously been considered as a rate limiting enzyme as its abundance and activity are highly regulated [39]. In the plant species where it has been examined, both ACS and ACO are encoded by multigene families, different members of which are expressed differentially in response to various developmental, environmental, and hormonal determinants. The *ACS* or *ACO* genes have been downregulated by transgenic approaches using antisense and shown to delay fruit ripening in climacteric species or senescence in flowers as reviewed in [40, 41]. Silencing the *ACO* gene has been shown to arrest ovule development in tobacco which is reversible with application of exogenous ethylene [42]. However, there are very few reports on how overexpression of the *ACO* gene affects the plant morphology. In *Arabidopsis*, formation of the apical hook during early seedling growth responds to ethylene by slowing growth of the cells leading to an exaggerated hook structure and serves as a model for ethylene responses [43]. Using in situ hybridization, expression of an *ACO* gene in the outer cells was correlated with the ethylene-induced exaggeration of the hook [44]. In addition to the *ACO* gene, three ethylene receptor/transporter genes (*ETR1*: Glyma.09G002600; *ETR2*: Glyma.10G188500 and *EIN4*: Glyma.10G008500) in the regulatory network of the ethylene response were differentially expressed in the minute-hilum seed coats (Fig. 3 and Table 3) at this same early stage of seed development as was an AP2/ERF (ethylene responsive factor).

Plant hormones, including ethylene, auxin, cytokinins, gibberellins, and abscisic acid influence each other and regulate in part the growth and development of seeds and pods [45]. Determining the regulatory role of ethylene has been complicated as it can have a biphasic dose-response curve with an optimum level that stimulates growth while higher or lower doses inhibit growth [46]. Recently, the molecular basis of *Semidwarf3,* a naturally occurring maize mutant, was shown to have elevated levels of ethylene due to a mutation creating an increase in stability of an ACC synthase gene. The shorter stature results from a decrease in cell elongation in the internode and concomitantly a promotion of cell elongation in the leaf auricle resulting in increased leaf angle [47]. An ACC deaminase transgenic line of canola which produces less ethylene than the plants from a wild type line, had smaller siliques with smaller and reduced number of seeds and also reduced levels of AUX and GA hormones [46].

A model for the postulated chain of events deduced from the gene expression changes found between the UC7 (*i*^*i*^ *R T Mi g*) and UC413 (*i*^*i*^ *R t mi G*) isolines was presented in Fig. 5. In the case of the UC413 *mi* line, overexpression of the *ACC oxidase* (*ACO*) gene, Glyma.09G008400, at early stages of pod and seed development could promote the release of higher levels of ethylene with a reciprocal decrease in AUXs and GAs and thus resulting also in the smaller hilum and possibly the smaller seed and pod sizes as shown in the *mi* isoline (Fig. 1, Table 1). We found that there were seven genes encoding Aux/IAA proteins (Table 4A) that were overexpressed in the *mi* isoline. Higher levels of Aux/IAA proteins often repress transcription of auxin-regulated genes such as auxin response factors (ARFs) with which they heterodimerize [23, 24]. Thus, expression of Aux/IAA proteins would potentially reduce the influence of auxin during development of the *mi* isoline.

In addition, a reduction in active GAs (GA1, GA4) could result from the increases in gibberellin 2-oxidase from the overexpressed genes, Glyma.07G236100 and Glyma.17G037300, in the *mi* isoline at both the EH10 and EH25 mg stages (Table 4B).

The gibberellin’s biosynthetic pathway and the enzymes involved in their synthesis and catabolism have been well studied in several plant species [48]. GA_1_ and GA_4_ appear to be the major bioactive GAs and they play a significant regulatory role in seed (seed coat, embryo) growth and development [49]. The two overexpressed gibberellin 2-oxidase genes in the *minute hilum* UC413 line, most likely catabolize and inactivate GA1 and GA4 as well as their precursors (GA9, GA12, GA20 and GA53) by 2β-hydroxylation [50]. Potentially, this could negatively affect expansion of the hilum or seed coat cells which occurs early in development leading to a smaller seed size. Since the seed coat also serves as channel to mobilize sugars and other phloem-derived nutrients to the developing embryo [51], a reduction in the size and weight of cotyledons and embryonic axis may also result.

Thus, the increased expression of the two *GA2ox4* genes brought upon by ethylene overproduction and consequent oxidation/inactivation of the bioactive GAs is a plausible network resulting in the reduction in active GAs leading to decreased cell growth and expansion during the embryo differentiation phase [50, 51]. These putative hormone disturbances caused by the higher ethylene content could cause the seed coat hilum region to mature faster or slow the seed coat cell growth in the hilum region. This would result in a smaller hilum. If the effects are manifested in tissues other than the seed coat, a higher ethylene might also result in inhibition of seed development and growth accounting for the smaller pods, number of seeds per pod, seed size and weight.

The hormonal unbalance created by the higher level of ethylene in the *mi* seeds likely results as well in the larger number of genes that are differentially expressed in several of the annotation categories. As shown in Fig. 2 and Table 2, these include the transcription factor, transcription related, protein metabolism, and photosynthesis related categories. The hormonal differences may have shifted the expression of some of these genes to be out of phase as compared to their normal pattern in the standard line. The lower GA levels induced by the higher ethylene content in the developing seeds of the *mi* isoline could in turn affect the expression of gibberellin regulated cell wall proteins such as extensin and other proline-rich proteins (as SbPRP2 and SbPRP3) which then exacerbate the cracking of the seed coat as observed in the minute hilum seeds. Previous studies have demonstrated correlation of soybean seed coat defectiveness of the recessive *i t* genotype combination (pigmented and defective) or with the *def* gene that specifies a net-like seed coat appearance [13–15].

For the allele combination of the minute hilum isoline UC413 (*i*^*i*^ *R t mi G*) compared to the UC7 (*i*^*i*^ *R T Mi g*) standard isoline, we report here a much higher number (90) of cell wall related genes that were overexpressed in the *mi* line versus in the standard line at the early EH10 stage, suggesting an importance of cell wall proteins in mediating the defective seed coat phenotype. In *mi* seed, the cracking appears to originate near the hilum at a later stage of development as the seed begins to desiccate. Since the cracks occur in the yellow area of the seed coat, this pattern appears to be different from the epistatic interaction of the recessive *i* and *t* alleles in which cracks are only found on the pigmented regions of the seed coat.

On the other hand, very few cell wall related genes were overexpressed in the UC7 standard line at the EH10 and EH25 stages (Table 2, Fig. 4) but more were overexpressed in the later stages. Among these are several Glyma models, originally in the hormone category, and annotated as gibberellin-regulated extensin genes (Additional file 4). In addition, another highly expressed cell wall gene, *SbPRP3* (Glyma.11G154900), was found to be overexpressed in the standard line (1005 RPKMs) compared to the *mi* line (287 RPKMs) only at the third stage of seed coat examined, EH50, along with four additional putative extensin genes (Additional file 4).

The fact that larger numbers of cell wall related genes were overexpressed in the *mi* seed coats, many encoding cell wall structural and loosening enzymes (cellulases, glycosyl hydrolases, expansins, chitinases, pectin lyases) resembles expression patterns in ethylene induced senescence or fruit ripening events [45]. At the same time, the lower expression in the minute hilum seeds of the gibberellin-regulated extensin genes, and the lower expression of *SbPRP3* (Gyma.11G154900) and other hydroxyproline rich proteins in the EH50 stage might contribute to limiting cell wall growth and perhaps enhancing the seed coat cracking, as manifested in the *mi* seeds during the desiccation phase later in development.

## Conclusions

The differences in gene expression profiles captured from the hilum and immediate surrounding region of the seed coat, at three early stages of seed development, supported a model for a series of events driven by higher levels of ethylene in the minute hilum (*mi*) isoline which had heretofore never been investigated except for isoline development. Overexpression of the ethylene-forming enzyme ACC Oxidase (*ACO*) gene, Glyma.09G008400, that catalyzes the final step in the biosynthesis of ethylene along with increased expression of three ethylene receptor/transporter genes (*ETR1*: Glyma.09G002600; *ETR2*: Glyma.10G188500 and EIN4: Glyma.10G008500) is predicted to result in higher levels of ethylene in the *mi* seed coat tissue. Higher levels of ethylene bring about lower levels of auxin (IAA) and gibberellins (GAs). These hormonal changes were supported by the higher expression of seven genes encoding Aux/IAA proteins, potential repressors of auxin-regulated transcriptional activation, and the two gibberellin 2-oxidase genes, which will induce a reduction in active GAs (GA1, GA4). The lower auxin levels and active gibberellins would affect cell wall metabolism and composition to decrease cell growth and expansion, and possibly speed maturation of the hilum region during the embryo differentiation phase, explaining the smaller hilum and seed phenotype in the *minute hilum* isoline. Since no structural differences were identified in Glyma.09G008400 between the two isolines, the *ACO* gene is not likely to be the *mi* gene itself but to manifest the phenotype through perturbation of its regulation or expression during development of the hilum in the seed coat. Comparative analyses of whole genome resequencing data combined with the differentially expressed gene sets of the two isolines revealed a relatively small number of Glyma models with potential insertions or deletions in their protein coding regions. These genes form a set of potential candidate genes for further investigation to determine the molecular nature of the *mi* gene.

## Methods

### Plant material and genotypes

The *Glycine max* lines used in this study were obtained from the United States Department of Agriculture/Agricultural Research Service, USDA/ARS soybean germplasm collection in Urbana, Illinois. The standard Clark 63 (UC7, PI 548532) and the mutant isoline UC413 (PI 547628) that was generated after the *mi G t* alleles of a line derived from a cross between the varieties Laredo and Harosoy were introgressed into the Clark 63 (UC7) standard after six backcrosses. The UC7 standard Clark 63 line has a *i*^*i*^ *R T Mi g* genotype and black hilum on yellow seed coat phenotype while the mutant isoline, UC413, has an *i*^*i*^ *R t mi G* genotype and a smaller hilum known as a minute hilum designated as the *mi* gene [38]. In the presence of the recessive *t* allele, the hilum is light in color (imperfect black) in the *mi* seed. The light green seed coat of the UC413 mutant seed at maturity is attributed to the dominant *G* locus (Fig. 1). UC numbers are short internal laboratory numbers. Additional file 1 lists all information about the seed used including Plant Introduction (PI) numbers that can be used to search for the information that is available from the GRIN (Germplasm Resources Information Network at https://www.ars-grin.gov/) maintained by the USDA/ARS and from which the seed can be obtained.

### RNA extraction, high-throughput RNA sequencing and alignment to the *Glycine max* gene models

Seed coat sections containing the hilum and a small portion of proximal surrounding area were dissected (here designated as the extended hilum EH region) from seed coats of the standard UC7 and the mutant UC413 genotypes as indicated by the white oval line in Fig. 1. The seed coat hilum regions were dissected at three early stages of seed maturation based on the fresh weight (fwt) of the whole seed and designated EH10 (10–25 mg fwt), EH25 (25–50 mg fwt) and EH50 50–100 mg fwt) with two independent replicates each.

Total RNA was isolated from those 12 groups of the extended hilum region using a phenol-chloroform and lithium chloride precipitation method as described [9]. The purified RNA samples were sequenced at the W. M. Keck Center for Comparative and Functional Genomics at the University of Illinois at Urbana-Champaign. Starting with one μg total RNA per sample, RNAseq libraries were constructed with the TruSeq RNA Sample Preparation Kit (Illumina San Diego, CA). These libraries were multiplexed and loaded onto 8-lane flowcells for cluster formation and sequenced on an Illumina HiSeq2000. One of the lanes was loaded with a PhiX Control library that provides a balanced genome for calculation of matrix, phasing and prephasing, which are essential for accurate basecalling. The libraries were sequenced from one end (single-reads) of the molecules to a total read length of 100-nt. The sequencing run generated.bcl files were converted into demultiplexed compressed fastq files using Casava 1.8.2 (Illumina, San Diego, CA). A secondary pipeline decompressed the fastq files, generated plots with quality scores using FastX Tool Kit, removed perfect matches to reads that contain only adaptor and generated a report with the number of reads per sample/library.

Information on the libraries is shown in Additional file 1. Also shown in Additional file 1, are single samples of several other RNA-Seq libraries that were constructed and analyzed from EH100 (extended hilum region of 100–200 mg seed) as well as the whole seed coat of the 10–20 mg seed (designated WS10), 25–50 mg seed (WS25), and 50–100 mg seed (WS50).

### RNA-Seq alignment and data normalization

The resulting sequence reads were aligned to the 88,647 Glyma cDNA soybean gene models determined by the Soybean Genome Project, Department of Energy, Joint Genome Institute (JGI/Phytozome) [17], using the alignment program Bowtie v1 [18]. Bowtie parameters allowed up to three mismatches and up to 25 alignments per read; reads aligned more than 25 times were discarded, with none of their alignments added to the final count. The total read counts, generally from 49 to 59 million per sample are shown in Additional file 1.

RNA-Seq data were normalized in reads per kilobase of gene model per million mapped reads (RPKM), since the RNA-Seq reads will map all through the length of a given transcript [19].

The annotations of Glyma gene models were downloaded from the soybean genome in Phytozome version 11.0 for the Wm82.a2 assembly [17]. Our annotation grouping into approximately 24 categories were completed manually using the PFAM and best Arabidopsis hit information from the GFF files. DESeq (available via Bioconductor) is an R package to analyze the resulting number of counts from high-throughput sequencing data and determine differential expression between different samples [52]. The basemean value and *p* values and adjusted *p* value (*padj*) for each of the 88,647 Glyma model were determined using this R package in comparison between Clark standard (UC7) and the minute hilum (*mi*) mutant line (UC413).

### Construction of genomic DNA libraries and sequencing on the Illumina HiSeq2000

Shotgun genomic DNA libraries were constructed by the DNA Sequencing Center, University of Illinois, using the TruSeq DNA Sample prep kit (San Diego, CA) as described previously [7]. The libraries were loaded onto separate flowcells and sequenced on an Illumina HiSeq2000. The DNAs in these libraries were sequenced from both ends of the molecules to a total read length of 100 nt from each end using a TruSeq SBS sequencing kit version 3. The run generated raw basecall files (.bcl) which were converted into demultiplexed compressed fastq files using Casava 1.8 (Illumina, CA). The total numbers of read counts of approximately 200 million at each end are shown in Additional file 1. Alignments using Bowtie v2 to the Wm82.a2 assembly and displayed using the Integrative Genomics Viewer were also performed as previously indicated [7]. In house python scripting was used to match VCF position information from the alignment data to Glyma models and expression data.

### Amplicon sequencing

Amplicon sequencing was conducted following PCR conditions as previously described [6]. The primers used to amplify the *ACO* gene were:

Forward primer ACO3F: 5’GTCATCTGCATGAGATCGAAACCTCCAAG3′ and Reverse primer ACO4R: 5’CCAAACGTGTCTATTAATGTAATTAAG3’. For each soybean genotype, four reactions were pooled and purified with a Zymo DNA Clean and Concentrator kit and the concentration adjusted to between 40 to 64 ng/ul. Samples were submitted for sequencing with the Illumina MySeq, and automated assembly by the Center for Computational and Integrative Biology DNA Core Facility at Massachusetts General Hospital (Cambridge, MA). Coverage of each amplicon was from 84,000 to over 168,000 reads. Multiple alignments were conducted with Multalin [53].

## Supplementary information

**Additional file 1.** Summary of information for cultivars, RNA-Seq, DNA genomic resequencing, and amplicon sequencing used in this report.

**Additional file 2 **RNASeq data analysis for 3786 Glyma models differentially expressed in the extended hilum regions between the standard UC7 (*i*^*i*^ *R T Mi g*) and minute hilum isolines UC413 (*i*^*i*^ *R t mi G*).

**Additional file 3 **Expression levels of ethylene forming enzyme gene (*ACO*) family members based on RNA-Seq reads aligning to the transcript sequence of Williams 82.

**Additional file 4.** Selected Glyma models of differentially expressed cell-wall protein or related genes in the three stages of development.

**Additional file 5.** Overexpressed transcription factors in either UC7 or UC413 isolines.

**Additional file 6.** Expression analysis of genes associated with seed size in soybean or other plant species.

**Additional file 7 **Protein alignments of four ACO oxidases from soybean against *Arabidopsis* and tomato proteins using the Multalin tool.

**Additional file 8.** Comparison of RPKMs for 11 Glyma models annotated as ACO genes in Williams.

**Additional file 9 **VCF file data of differences in the ACO (Glyma.09G008400.1) genomic sequences of alleles from UC7 *(i*^*i*^ *R T)* and UC413 *(i*^*i*^ *R t mi G)* lines.

**Additional File 10.** Alignments of ACO gene (Glyma.09G008400.1) promoter regions derived from amplicon sequences UC7 and UC413 alleles.

**Additional file 11.** Coverage graphs and amplicon raw sequencing data.

**Additional file 12 **Genomic sequence alignments of the *R* and *T* loci for the UC2 (*R T*), UC7 (*R T*), UC413 (*R t*) and UC501 (*r t*) soybean lines.

**Additional file 13.** Matching of sequence variation with RNA-Seq data in UC7 and UC413.

## Data Availability

The RNA-Seq datasets supporting the conclusions of this article are available in the Gene Expression Omnibus at the National Center for Biotechnology Information with Accession Series GSE146287 at (https://www.ncbi.nlm.nih.gov/geo/). The genomic resequencing datasets from the Short Read Archive (https://www.ncbi.nlm.nih.gov/sra) supporting the conclusions of this article are shown in Additional file 1 and amplicon sequencing data supporting the conclusions of this article are shown in Additional file 11 of this article.

## Abbreviations

*ACO*: 1-aminocyclopropane-1-carboxylate oxidase = ‘ethylene forming enzyme’ gene
ABA: Abscisic acid
AUX: Auxin
CHS: Chalcone synthase
CK: Cytokinins
DAP: Days after pollination
AUX: Auxin
fwt: Fresh weight
F3’H: Flavonoid 3′ hydroxylase
GA: Gibberellins
*padj*: Adjusted *p value* = statistical significance term
*pval*: *p* value = statistical significance term
RPKM: Reads per kilo base per million mapped reads
RPKM: Reads per kilo base per million mapped reads
*UF3G*: Flavonoid 3-Oglucosyltransferase

## References

[1] Carlson JB, Lersten NR: Reproductive morphology. In Boerma HR, Specht JE, editors. Soybeans: improvement, production, and uses. Madison WI. 2004; American Society of Agronomy. pp. 59–95.

[2] Smýkal P, Vernoud V, Blair MW, Soukup A, Thompson RD (2014). The role of the testa during development and in establishment of dormancy of the legume seed. Front Plant Sci.

[3] Todd JJ, Vodkin LO (1996). Duplications that suppress and deletions that restore expression from a CHS multigene family. Plant Cell.

[4] Tuteja JH, Zabala G, Varala K, Hudson M, Vodkin LO (2009). Endogenous, tissue-specific short interfering RNAs silence the chalcone synthase gene family in Glycine max seed coats. Plant Cell.

[5] Cho YB, Jones SI, Vodkin LO (2013). The transition from primary siRNAs to amplified secondary siRNAs that regulate chalcone synthase during development of *Glycine max* seed coats. PLoS One.

[6] Cho YB, Jones SI, Vodkin LO (2017). Mutations in *Argonaute5* illuminate Epistatic interactions of the *K1* and *I* loci leading to saddle seed color patterns in *Glycine max*. Plant Cell.

[7] Cho YB, Jones SI, Vodkin LO (2019). Nonallelic homologous recombination events responsible for copy number variation within an RNA silencing locus. Plant Direct.

[8] Toda K, Yang D, Yamanaka N, Watanabe S, Harada K, Takahashi R (2002). A single-base deletion in soybean flavonoid 3′-hydroxylase gene is associated with gray pubescence color. Plant Mol Biol.

[9] Zabala G, Vodkin L (2003). Cloning of the pleitropic *T* locus in soybean and two recessive alleles that differentially affect structure and expression of the encoded flavonoid 3’ hydroxylase. Genetics..

[10] Gillman JD, Tetlow A, Lee J-D, Shannon JG, Bilyeu K (2011). Loss-of-functions affecting a specific Glycine max R2R3 MYB transcription factor result in Brown hilum and brown seed coats. BMC Plant Biol.

[11] Zabala G, Vodkin LO (2014). Methylation affects transposition and splicing of a large CACTA transposon from a MYB transcription factor regulating anthocyanin synthase genes in soybean seed coats. PLoS One.

[12] Nicholas CD, Lindstrom JT, Vodkin LO (1993). Variation of proline-rich cell wall proteins in soybean lines with anthocyanin mutations. Plant Mol Biol.

[13] Lindstrom JT, Vodkin LO (1991). A soybean Cell Wall protein 1s affected by seed color genotype. Plant Cell.

[14] Percy JD, Philip R, Vodkin LO (1999). A defective seed coat pattern (net) is correlated with the post-transcriptional abundance of soluble proline-rich cell wall proteins. Plant Mol Biol.

[15] Kour A, Boone AM, Vodkin LO (2014). RNA-Seq profiling of a defective seed coat mutation in glycine max reveals differential expression of proline-rich and other cell wall protein transcripts. PLoS One.

[16] Houben M, Van de Poel B (2019). 1-Aminocyclopropane-1-carboxylic acid oxidase (ACO): the enzyme that makes the plant hormone ethylene. Front Plant Sci..

[17] Schmutz J, Cannon SB, Schlueter J, Mitros T (2010). Genome sequence of the paleopolyploid soybean. Nature..

[18] Langmead B, Trapnell C, Pop M, Salzberg SL (2009). Ultrafast and memory efficient alignment of short DNA sequences to the human genome. Genome Biol.

[19] Mortazavi A, Williams BA, McCue K, Schaeffer L, Wold B (2008). Mapping and quantifying mammalian transcriptomes by RNA-Seq. Nat Methods.

[20] Vogel JP, Woeste KE, Theologis A, Kieber JJ (1998). Recessive and dominant mutations in the ethylene biosynthetic gene ACS5 of Arabidopsis confer cytokinin insensitivity and ethylene overproduction, respectively. PNAS..

[21] Chae HS, Faure F, Kieber JJ (2003). The *eto1*, *eto2*, and *eto3* mutations and cytokinin treatment increase ethylene biosynthesis in Arabidopsis by increasing the stability of ACS protein. Plant Cell.

[22] Wang KL-C, Li H, Ecker JR (2002). Ethylene biosynthesis and signaling networks. Plant Cell.

[23] Overvoorde PJ, Okushima Y, Alonso JM, Chan A, Chang C, Ecker JR, Hughes B, Liu A, Onodera C, Quach H, Smith A, Yu G, Theologis A (2005). Functional genomic analysis of the AUXIN/INDOLE-3-ACETICACID gene family members in Arabidopsis thaliana. Plant Cell.

[24] Ren H, Gray WM (2015). SAUR proteins as effectors of hormonal and environmental signals in plant growth. Mol Plant.

[25] Nawaz MA, Rehman HM, Imtiaz M, Baloch FS, Lee JD, Yang SH, Lee SI, Chung G (2017). Systems identification and characterization of cell wall reassembly and degradation related genes in *Glycine max* (L.) Merill, a bioenergy legume. Sci Rep.

[26] Hong JC, Nagao RT, Key JL (1990). Characterization of a proline-rich cell wall protein gene family of soybean. A comparative analysis. J Biol Chem.

[27] Ge L, Yub J, Wanga H, Luthc D, Baib G, Wangc K, Chena R (2016). Increasing seed size and quality by manipulating *BIG SEEDS1* in legume species. PNAS..

[28] Liu L, Tong H, Xiao Y, Che R, Xu F, Hu B, Liang C, Chu J, Li J, Chu C (2015). Activation of big Grain1 significantly improves grain size by regulating auxin transport in rice. PNAS..

[29] Wang X, Li Y, Zhang H, Sun G, Zhang W, Qiu L (2015). Evolution and association analysis of *GmCYP78A10* gene with seed size/weight and pod number in soybean. Mol Biol Rep.

[30] Zhao B, Dai A, Wei H, Yang S, Wang B, Jiang N, Feng X (2016). *Arabidopsis KLU* homologue *GmCYP78A72* regulates seed size in soybean. Plant Mol Biol.

[31] Du J, Wang S, He C, Zhou B, Ruan J-W, Shou H (2017). Identification of regulatory networks and hub genes controlling soybean seed set and size using RNA sequencing analysis. J Exp Bot.

[32] Jones SI, Vodkin LO (2013). Using RNA-Seq to profile soybean seed development from fertilization to maturity. PLoS One.

[33] Shamimuzzaman M, Vodkin LO (2014). Transcription factors and glyoxylate cycle genes prominent in the transition of soybean cotyledons to the first functional leaves of the seedling. Func Intr Genomics.

[34] Langmead B, Salzberg SL (2012). Fast gapped-read alignment with bowtie 2. Nat Methods.

[35] Solovyev VV, Shahmuradov IA, Salamov AA (2010). Identification of promoter regions and regulatory sites. Methods Mol Biol.

[36] Shahmuradov I (2015). Solovyev V: Nsite, NsiteH and NsiteM computer tools for studying transcription regulatory elements. Bioinformatics..

[37] Cominelli E, Galbiati M, Albertini A, Fornara F, Conti L, Coupland G, Tonelli C (2011). DOF-binding sites additively contribute to guard cell-specificity of *AtMYB60* promoter. BMC Plant Biol.

[38] Waddle BA (1960). The inheritance of minute hilum in soybeans.

[39] Kende H (1993). Ethylene biosynthesis. Annu Rev Plant Physiol Plant Mol Biol.

[40] Sterns JC, Glick BR (2003). Transgenic plants with altered ethylene biosynthesis or perception. Biotechnol Adv.

[41] Phillips AL: Genetic and transgenic approaches to improving crop performance. In: Davies P.J. (eds) Plant hormones. 2010;618-645, Springer, Dordrecht.

[42] De Martinis D, Mariani C (1999). Silencing gene expression of the ethylene-forming enzyme results in a reversible inhibition of ovule development in transgenic tobacco plants. Plant Cell.

[43] Schaller GE, Kieber JJ: Ethylene. In the Arabidopsis book. 2002;Amer. Soc Plant Biol. 10.1199/tab.0071 PMC3243340 22303216

[44] Raz V, Ecker JR (1999). Regulation of differential growth in the apical hook of *Arabidopsis*. Development..

[45] Iqbal N, Kahn NA, Ferranto A, Trivellini A, Francini A, Khan MIR (2017). Ethylene role in plant growth, development and senescence: interaction with other phytohormones. Front Plant Sci.

[46] Walton LJ, Kurepin LV, Yeung EC, Shah S, Emery RJN, Reid DM, Pharis RP (2012). Ethylene involvement in silique and seed development of canola, *Brassica napus* L. Plant Physiol Biochem.

[47] Li H, Wang L, Liu M, Dong Z, Li Q, Fei S, Xiang H, Liu B, Jin W (2020). Maize plant architecture is regulated by the ethylene biosynthetic gene *ZmACS7*. Plant Physiol.

[48] Hedden P, Thomas SG (2012). Gibberellin biosynthesis and its regulation. Biochem J.

[49] Nadeau CD, Ozga JA, Kurepin LV, Jin A, Pharis RP, Reinecke DM (2011). Tissue-specific regulation of gibberellin biosynthesis in developing pea seeds. Plant Phys.

[50] Lee DH, Lee IC, Kim KJ, Kim DS, Na HJ, Lee IJ, Kang SM, Jeon HW, Le PY, Ko JH (2014). Expression of gibberellin 2-oxidase 4 from *Arabidopsis* under the control of a senescence-associated promoter results in a dominant semi-dwarf plant with Normal flowering. J Plant Biol.

[51] Van Dongen JT, Ammerlaan AM, Wouterlood M, Van Aelst AC, Borstlap AC (2003). Structure of the developing pea seed coat and the post phloem transport pathway of nutrients. Ann Bot (Lond).

[52] Anders S, Huber W (2010). Differential expression analysis for sequence count data. Genome Biol.

[53] Corpet F (1988). Multiple sequence alignment with hierarchical clustering. Nucl Acids Res.

