# Supplementary material for: Overexpression of an ethylene-forming ACC oxidase (ACO) gene precedes the Minute Hilum seed coat phenotype in Glycine max
Source: BMC Genomics. 2020 Oct 16;21:716. doi: 10.1186/s12864-020-07130-8 (PMC7566151; doi:10.1186/s12864-020-07130-8)

Additional file 3. **Expression levels of ethylene forming enzyme gene (*ACO*) family members based on RNA-Seq reads aligning to the model transcript sequence of Williams 82.** Alignments of RNA-Seq reads from the EH10 stage of the UC413 isoline (i^i *R t mi G*) to the gene model transcripts did not allow for any mismatches. X-axis is position along the transcript and Y-axis is RPKM level.

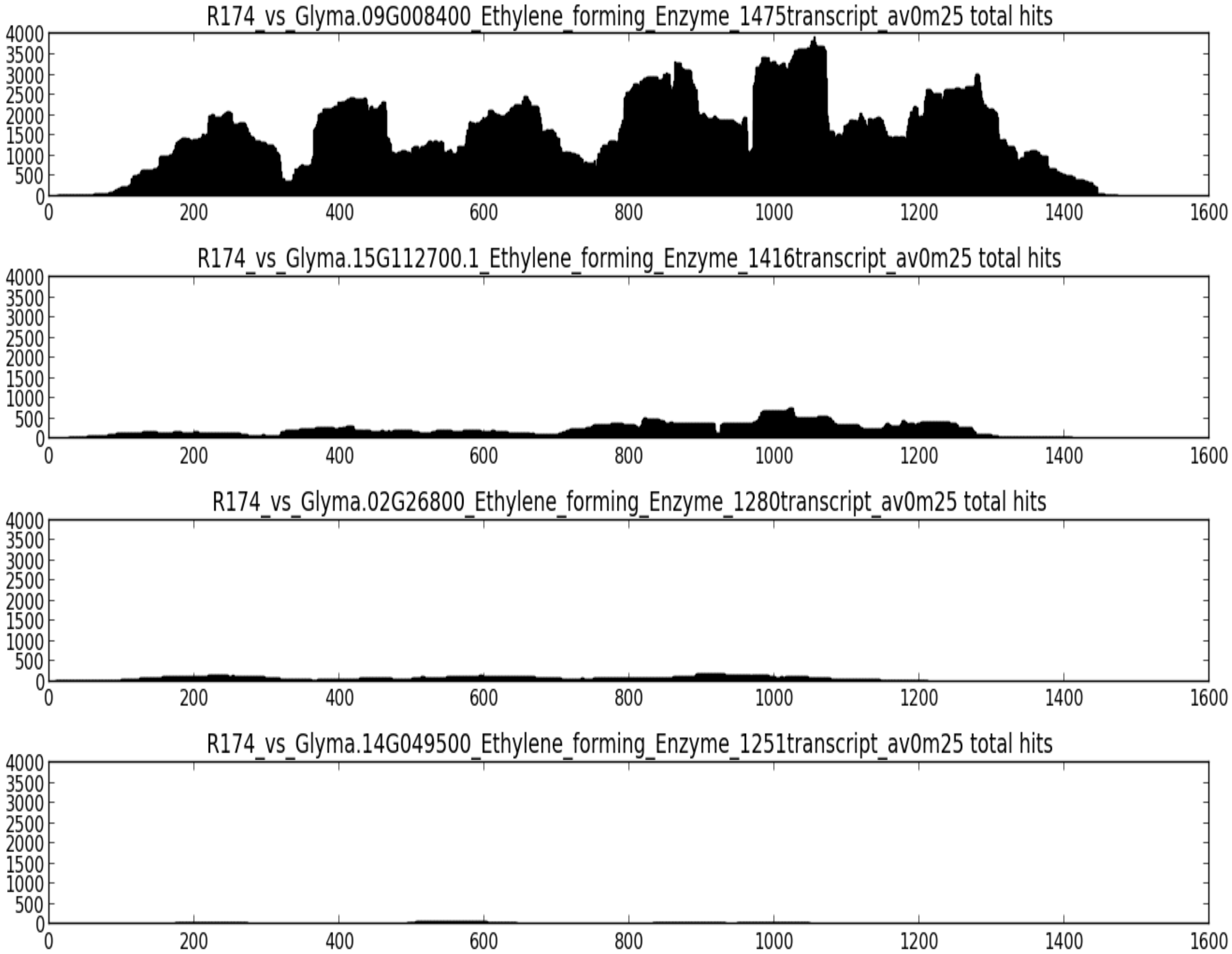

Supplement: Supplementary file 3 — Additional file 3 Expression levels of ethylene forming enzyme gene (ACO) family members based on RNA-Seq reads aligning to the transcript sequence of Williams 82. [file 12864_2020_7130_MOESM3_ESM.pdf]
