# Supplementary material for: Overexpression of an ethylene-forming ACC oxidase (ACO) gene precedes the Minute Hilum seed coat phenotype in Glycine max
Source: BMC Genomics. 2020 Oct 16;21:716. doi: 10.1186/s12864-020-07130-8 (PMC7566151; doi:10.1186/s12864-020-07130-8)
Supplement: Supplementary file 6 — Additional file 6. Expression analysis of genes associated with seed size in soybean or other plant species. [file 12864_2020_7130_MOESM6_ESM.pdf]

Additional file 6: Expression analysis of genes associated with seed size in soybean or other plant species.

Glyma Model	Annotation	Gene	Reference & Species	Ave RPKMs UC7	Ave RPKMs UC413	Fold Change	Seed Stage
Glyma.10G244400.1	TIFY domain /Divergent CCT motif	***GmBS1* (*AtPPD2*)**	Ge.., 2016 (*Mt, Gm*)	No differential expression any stage			
Glyma.20G150000.1	TIFY domain /Divergent CCT motif	***GmBS2* (*AtPPD1*)**	"	No differential expression any stage			
Glyma.10G208200.1	U-Box Domain-Containing Protein 14	***GmBS***	"	No differential expression any stage			
Glyma.16G006100.1	BIG GRAIN 1-like B; Aux transport reg	***BG1*-like**	Liu..,2015 (*Os*)	No differential expression any stage			
Glyma.07G036700.1[a]	BIG GRAIN 1-like B; Aux transport reg	***BG1*-like**	"	4.33	**9.88**	0.44	EH10
Glyma.16G158700.1	Chaperone-Activity Of Bc1 Complex	***ABC1*-like**	Li.., 2015 (*Os*)	2.97	**8.90**	0.33	EH10
Glyma.16G158700.1	Chaperone-Activity Of Bc1 Complex	***ABC1*-like**	"	1.72	**4.26**	0.40	EH25
Glyma.03G176600.2	WRKY family TF	***TTG2***	Johnson..,2002 (*At*)	11.75	**[illegible]3.06**	0.36	EH10
Glyma.03G176600.1	WRKY family TF	***TTG2***	"	14.1	**[illegible]0.81**	0.46	EH25
Glyma.05G091200.1	Integrase-type DNA-binding	***AP2***	Jofuki..,2005 (*At*)	2.83	**7.19**	0.39	EH50
Glyma.16G142700.1	LIM domain-containing protein	***DA1***	Xia..,2013 (*At*)	1.48	**4.24**	0.35	EH10
Glyma.10G137600.1	ABC TRANSPORTER B 11	***PGP4***	Peer..,2007 (At)	2.97	**8.90**	0.33	EH10
Glyma.02G008000.1	ABC TRANSPORTER B 11	***PGP4***	"	4.14	**[illegible]3.63**	0.30	EH10
Glyma.15G208600.1	Transporter: Auxin efflux carrier	***GmPIN2b***	Liu & Wei. 2017 (*Gm*)	1.96	**6.65**	0.29	EH10
Glyma.05G019200.1	CYT P450 78A5-RELATED	***KLU***	Wang..2015 ; Zhao 2016	0.81	**5.11**	0.16	EH10
Glyma.05G019200.1	CYT P450 78A5-RELATED	***KLU***	Du.., 2017 (*Gm*)	2.17	**6.83**	0.32	EH25
Glyma.16G021200.1	CYT P450 78A6-RELATED	***CYP78A6***	Zhao.., 2016 (*Gm*)	**5.21**	1.37	3.80	EH10
Glyma.16G021200.1	CYT P450 78A6-RELATED	***CYP78A6***	"	**15.09**	3.23	4.67	EH25
Glyma.16G021200.1	CYT P450 78A6-RELATED	***CYP78A6***	"	**10.31**	2.24	4.60	EH50
Glyma.07G052300.1	CYT P450 78A6-RELATED	***CYP78A6***	"	**5.74**	1.72	3.34	EH25
Glyma.07G052300.1	CYT P450 78A6-RELATED	***CYP78A6***	"	**2.10**	0.68	3.09	EH50
Glyma.06G202300.1[b]	Flavonoid 3'-Monooxygenase	***T* (*F3'H*)**	Zabala.., 2003 (*Gm*)	**108.5**	42.3	2.55	EH50

The average RPKMs for both repeats of each isoline is shown to conserve space. See Additional File 2 for both values. FC= fold change of UC7/UC413 RPKMs. Bold values show the higher expression level. The gene name is that given in the indicated reference. Glyma models with homology were determined by sequence similarity. *Mt, Medicago truncatula; At, Arabidopsis thalina; Os, Oryza sativa; Gm, Glycine max*

[a] The differential expression of this gene between the standard UC7 and the mutant UC413 lines had *pval*<0.05 but higher *padj*.

[b] This Glyma model is not associated with seed size but encodes the soybean *T* locus and has a known premature stop codon in the recessive *t* allele found in the *mi* isoline that affects transcript levels.

References:

Doughty J, Aljabri M, Scott RJ: Flavonoids and the regulation of seed size in *Arabidopsis*. Biochemical Society Transactions. 2014;42 (2):364-369. (review)

Du J, Wang S, He C, Zhou B, Ruan J-W, Shou H: Identification of regulatory networks and hub genes controlling soybean seed set and size using RNA sequencing analysis. Journal of Experimental Botany. 2017;68 (8):1955–1972.

Ge L, Yub J, Wanga H, Luth D, Baib G, Wangc K, Chena R: Increasing seed size and quality by manipulating *BIG SEEDS1* in legume species. PNAS. 2016;113(44):12414-12419.

Jofuku KD, Omidyar PK, Gee Z, Okamuro K: Control of seed mass and seed yield by the floral homeotic gene *APETALA2*. PNAS. 2005;102:3117-3122.

Johnson CS, Kolevski B, Smyth DR: *TRANSPARENT TESTA GLABRA2*, a trichome and seed coat development gene of Arabidopsis, encodes a WRKY transcription factor. Plant Cell. 2002;14 (6):1359-1375.

Li T, Jiang J, Zhang S, Shu H, Wang Y, Lai J, Du J, Yang C: *OsAGSW1*, an ABC1-like kinase gene, is involved in the regulation of grain size and weight in rice. Journal of Experimental Botany. 2015; 66 (19):5691 –5701.

Liu L, Tong H, Xiao Y, Che R, Xu F, Hu B, Liang C, Chu J, Li J, Chu C: Activation of Big Grain1 significantly improves grain size by regulating auxin transport in rice. PNAS. 2015;112(35):11102-11107.

Liu Y, Wei H: Genome-wide identification and evolution of the *PIN-FORMED* (*PIN*) gene family in *Glycine max.* Genome 2017, 60:564–571.

Peer WA, Murphy AS: Flavonoids and auxin transport: modulators or regulators? TRENDS in Plant Science. 2007;12:557-563.

Wang X, Li Y, Zhang H, Sun G, Zhang W, Qiu L: Evolution and association analysis of *GmCYP78A10* gene with seed size/weight and pod number in soybean. Mol,Biol. Reports. 2015;42:489-496.

Xia T, Li N, Dumenil J, Li J, Kamenski A, Bevan MW, Gao F, Li YH: The ubiquitin receptor *DA1* interacts with the E3 ubiquitin ligase *DA2* to regulate seed and organ size in *Arabidopsis*. Plant Cell. 2013;25:3347-3359

Zhao B, Dai A, Wei H, Yang S, Wang B, Jiang N, Feng X: *Arabidopsis KLU* homologue *GmCYP78A72* regulates seed size in soybean. Plant Molecular Biology. 2016;90:33-47.

Zabala G, Vodkin L: Cloning of the pleitropic *T* locus in soybean and two recessive alleles that differentially affect structure and expression of the encoded flavonoid 3' hydroxylase. Genetics. 2003;163:295-309.
