# Supplementary material for: Overexpression of an ethylene-forming ACC oxidase (ACO) gene precedes the Minute Hilum seed coat phenotype in Glycine max
Source: BMC Genomics. 2020 Oct 16;21:716. doi: 10.1186/s12864-020-07130-8 (PMC7566151; doi:10.1186/s12864-020-07130-8)

Additional file 7. Protein alignments of four ACO oxidases from soybean against *Arabidopsis* and tomato proteins using the Multalin tool.

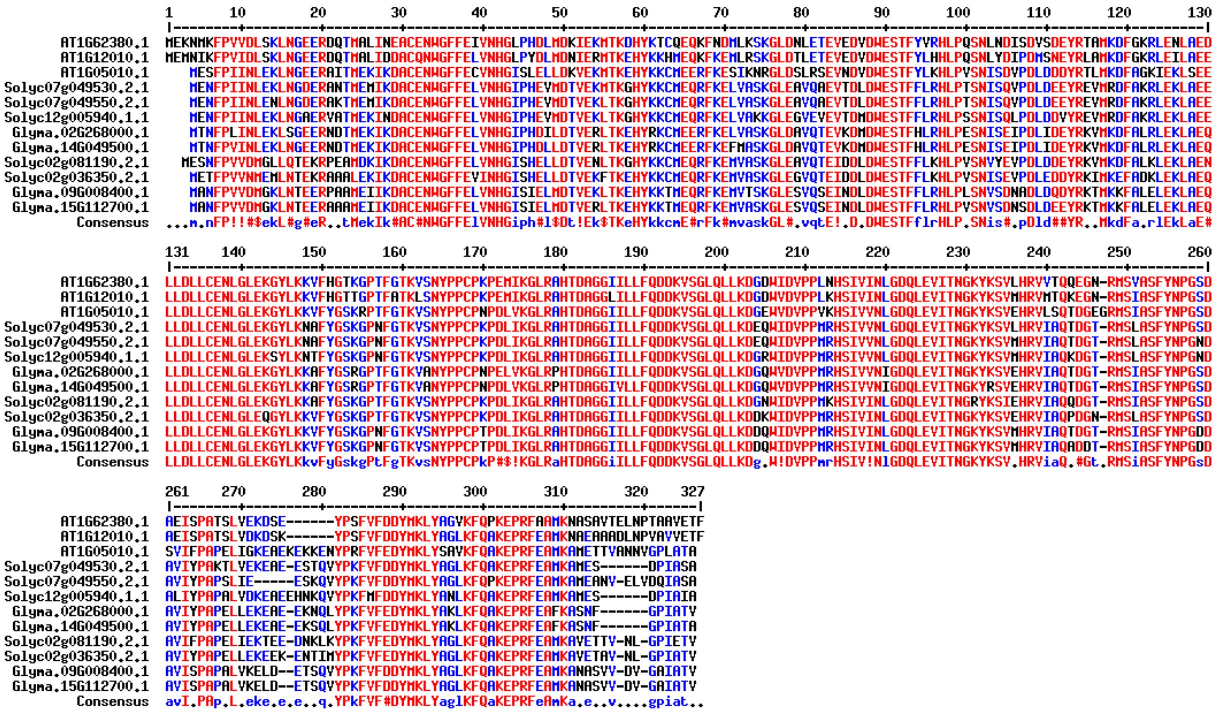

Supplement: Supplementary file 7 — Additional file 7 Protein alignments of four ACO oxidases from soybean against Arabidopsis and tomato proteins using the Multalin tool. [file 12864_2020_7130_MOESM7_ESM.pdf]
