# Supplementary material for: Overexpression of an ethylene-forming ACC oxidase (ACO) gene precedes the Minute Hilum seed coat phenotype in Glycine max
Source: BMC Genomics. 2020 Oct 16;21:716. doi: 10.1186/s12864-020-07130-8 (PMC7566151; doi:10.1186/s12864-020-07130-8)
Supplement: Supplementary file 9 — Additional file 9 VCF file data of differences in the ACO (Glyma.09G008400.1) genomic sequences of alleles from UC7 (ii R T) and UC413 (ii R t mi G) lines. [file 12864_2020_7130_MOESM9_ESM.pdf]

Additional file 9. VCF file data of differences in the ACO genomic sequences of alleles from UC7 *(i^i R T)* and UC413 *(i^i R t mi G)* lines.

Source_File	#CHROM	POS	ID	REF	ALT
G037_UC7_VCF_extracted_range_Chr09_0_to_full.tsv	Chr09	662092	0	T	TTAAAGCA
G038_UC413_VCF_extracted_range_Chr09_0_to_full.tsv	Chr09	662092	0	TA	TTAAAGCAA,T
G038_UC413_VCF_extracted_range_Chr09_0_to_full.tsv	Chr09	662096	0	T	A
G038_UC413_VCF_extracted_range_Chr09_0_to_full.tsv	Chr09	662098	0	T	G
G038_UC413_VCF_extracted_range_Chr09_0_to_full.tsv	Chr09	662102	0	T	C
G037_UC7_VCF_extracted_range_Chr09_0_to_full.tsv	Chr09	662928	0	A	T
G038_UC413_VCF_extracted_range_Chr09_0_to_full.tsv	Chr09	662974	0	G	T

The VCF file from Bowtie genomic alignments are shown for ACO Glyma.09G008400. Ref is the Williams 82.a2 reference genome and ALT is the alternate base call as the position indicated (POS) in either the UC7 or UC413 genomic libraries.
