# Supplementary material for: Overexpression of an ethylene-forming ACC oxidase (ACO) gene precedes the Minute Hilum seed coat phenotype in Glycine max
Source: BMC Genomics. 2020 Oct 16;21:716. doi: 10.1186/s12864-020-07130-8 (PMC7566151; doi:10.1186/s12864-020-07130-8)
Supplement: Supplementary file 12 — Additional file 12 Genomic sequence alignments of the R and T loci for the UC2 (R T), UC7 (R T), UC413 (R t) and UC501 (r t) soybean lines. [file 12864_2020_7130_MOESM12_ESM.pdf]

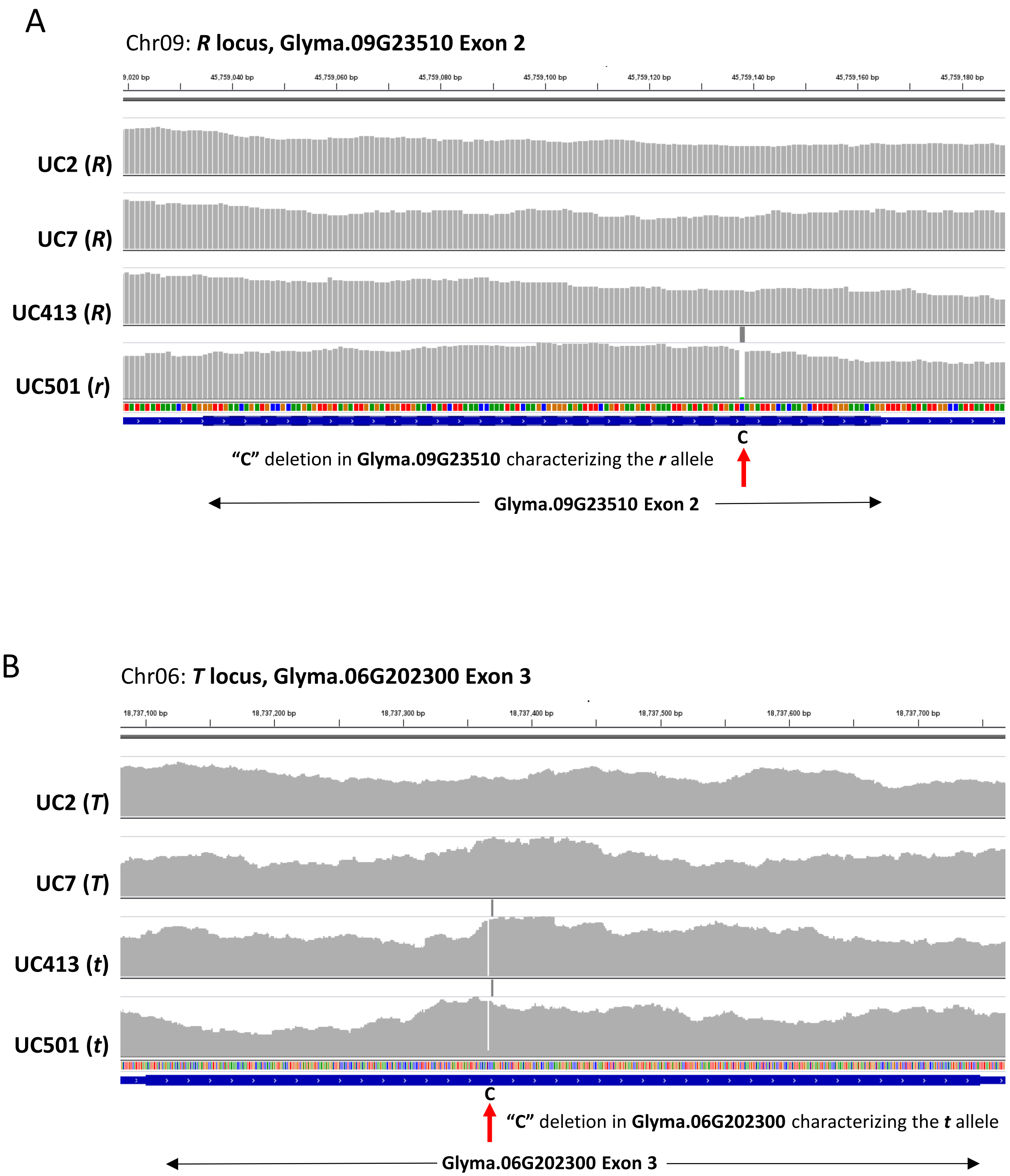

Additional file 12. Genomic sequence alignments of the *R* and *T* loci for the UC2 (*R T*), UC7 (*R T*), UC413 (*R t*) and UC501 (*r t*) soybean lines. A) The *R* locus, Glyma.09G23510 Exon 2 genomic sequence, showing the C deletion in UC501 (Harosoy) that characterizes the *r* allele. **B**) The *T* locus, Glyma.06G202300 Exon 3 genomic sequence, confirming the C deletion in both UC413 and UC501 characteristic of the *t* allele. Displays are presented from Bowtie 2 alignments with the Integrative Genomics Viewer.
