# Supplementary material for: Overexpression of an ethylene-forming ACC oxidase (ACO) gene precedes the Minute Hilum seed coat phenotype in Glycine max
Source: BMC Genomics. 2020 Oct 16;21:716. doi: 10.1186/s12864-020-07130-8 (PMC7566151; doi:10.1186/s12864-020-07130-8)

Additional file 10. Alignments of ACO gene (Glyma.09G008400.1) promoter regions derived from amplicon sequences UC7 and UC413 alleles. (Data used in Fig. 7) W82 is the genomic sequence of the 1505 bp Glyma.09G008400.1 region from the sequenced Williams 82 genome at Phytozome and W43 is the amplicon sequence of a closely related Williams isoline. Arrow represents the position where whole genome differences in the VCF file were found. Boxed sequence motifs mark the 5 WAAAG elements in the promoter.

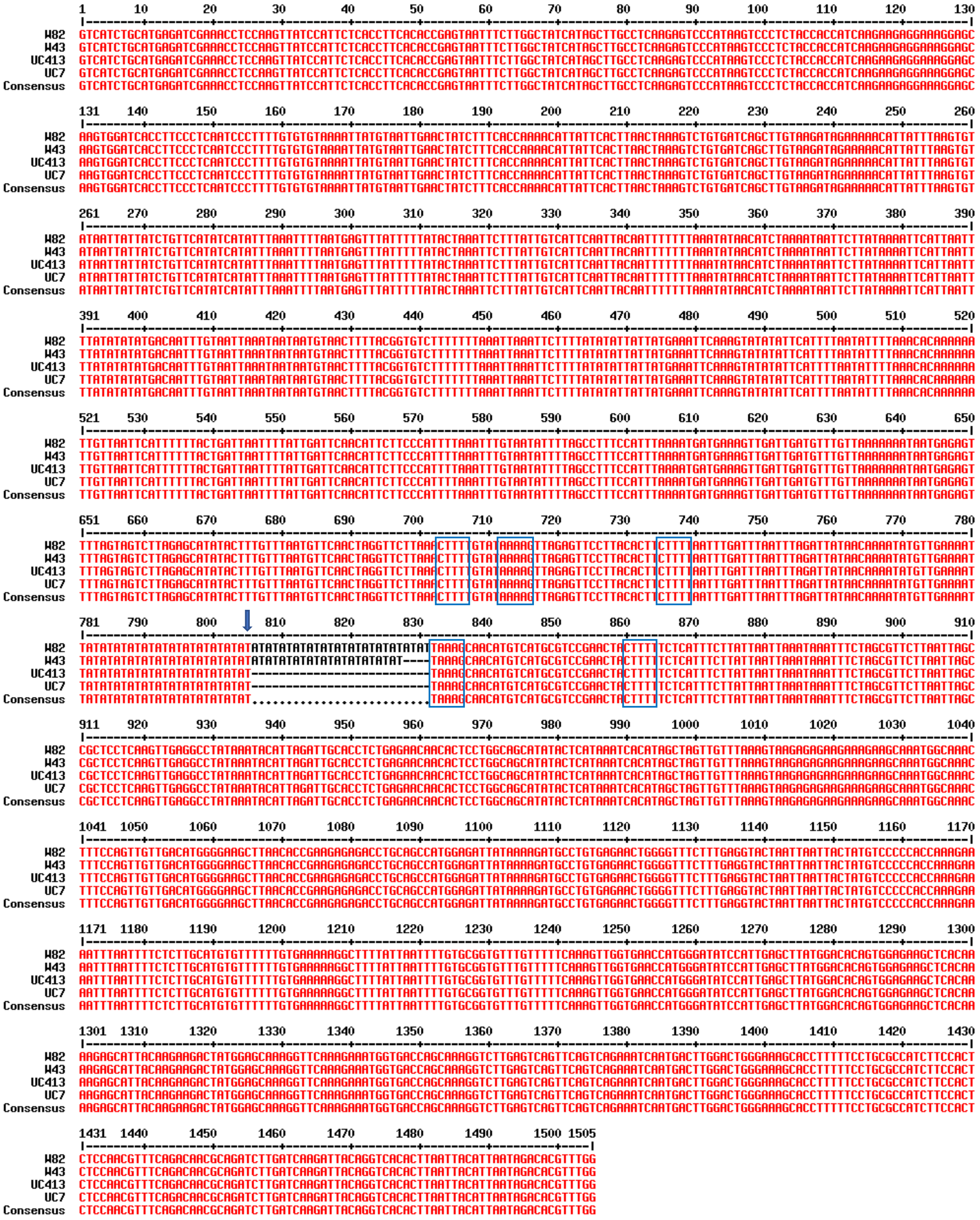

Supplement: Supplementary file 10 — Additional File 10. Alignments of ACO gene (Glyma.09G008400.1) promoter regions derived from amplicon sequences UC7 and UC413 alleles. [file 12864_2020_7130_MOESM10_ESM.pdf]
